# Using signal amplification by reversible exchange (SABRE) to hyperpolarise ^119^Sn and ^29^Si NMR nuclei†
†Data created during this research are available by request from the University of York Data Catalogue http://dx.doi.org/10.15124/12345.
‡
‡Electronic supplementary information (ESI) available: General procedures and experimental conditions, sample preparation, performing SABRE experiments, ligand exchange rates, field dependent polarisation transfer studies, synthetic methods. CCDC 1501636 and 1501637. For ESI and crystallographic data in CIF or other electronic format see DOI: 10.1039/c6cc07109k



**DOI:** 10.1039/c6cc07109k

**Published:** 2016-11-23

**Authors:** Alexandra M. Olaru, Alister Burt, Peter J. Rayner, Sam J. Hart, Adrian C. Whitwood, Gary G. R. Green, Simon B. Duckett

**Affiliations:** a Department of Chemistry , University of York , Heslington , York YO10 5DD , UK . Email: simon.duckett@york.ac.uk ; Tel: +44 1904 322564

## Abstract

The hyperpolarisation of the ^119^Sn and ^29^Si nuclei in 5-(tributylstannyl)pyrimidine (**A**_Sn_) and 5-(trimethylsilyl)pyrimidine (**B**_Si_) is achieved through their reaction with [IrCl(COD)(IMes)] (**1a**) or [IrCl(COD)(SIMes)] (**1b**) and *para*hydrogen *via* the SABRE process. **1a** exhibits superior activity in both cases. The two inequivalent pyrimidine proton environments of **A**_Sn_ readily yielded signal enhancements totalling ∼2300-fold in its ^1^H NMR spectrum at a field strength of 9.4 T, with the corresponding ^119^Sn signal being 700 times stronger than normal. In contrast, **B**_Si_ produced analogous ^1^H signal gains of ∼2400-fold and a ^29^Si signal that could be detected with a signal to noise ratio of 200 in a single scan. These sensitivity improvements allow NMR detection within seconds using micromole amounts of substrate and illustrate the analytical potential of this approach for high-sensitivity screening. Furthermore, after extended reaction times, a series of novel iridium trimers of general form [Ir(H)_2_Cl(NHC)(μ-pyrimidine-κ*N*:κ*N*′)]_3_ precipitate from these solutions whose identity was confirmed crystallographically for **B**_Si_.

## Abstract 2

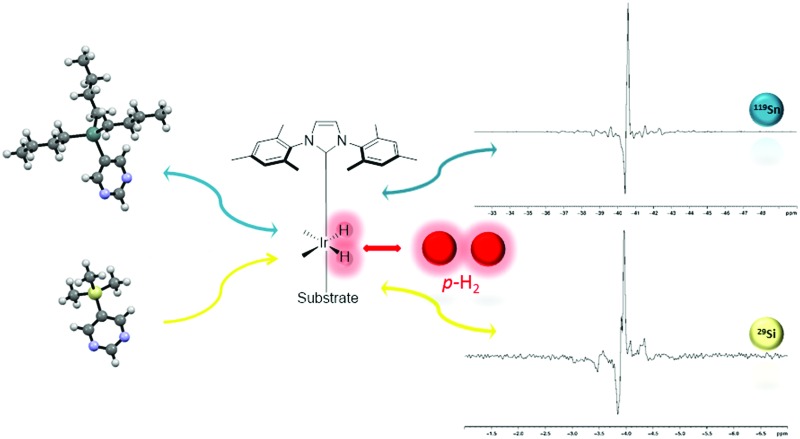
The hyperpolarisation of the ^119^Sn and ^29^Si nuclei in 5-(tributylstannyl)pyrimidine (**A**_Sn_) and 5-(trimethylsilyl)pyrimidine (**B**_Si_) is achieved through their reaction with [IrCl(COD)(IMes)] (**1a**) or [IrCl(COD)(SIMes)] (**1b**) and *para*hydrogen *via* the SABRE process.

Nuclear magnetic resonance (NMR) spectroscopy is an incredibly powerful technique which yields chemically diagnostic information that is highly useful for structure elucidation.^1^ It achieves this result despite suffering from inherently low sensitivity as a consequence of the fact that when atoms with a nuclear spin are placed in a magnetic field, their energy levels are only weakly split. While this Zeeman splitting increases with magnetic field strength, for standard NMR spectrometers the population difference, governed by the Boltzmann distribution, is very small. In fact, for protons at 298 K in a field of 9.4 T, the acquired signal is effectively derived from only 1 in every 32 000 of these nuclei, while the corresponding ^29^Si and ^119^Sn proportions are just 1 in ∼156 000 and ∼83 000 respectively. Their natural abundance of just 4.7 and 8.6% amplifies this effect even further.

Organotin reagents find wide use in synthetic chemistry in carbon–carbon sigma bond forming reactions that are often mediated by Pd(0) catalysts.^2^ This approach reflects a major advantage over traditional boronic acid methods due to the mild conditions that can be employed and the reaction's high functional group tolerance. Organosilanes react in a similar way with organohalides or triflates *via* the Hiyama coupling.^3^ They also find use as protecting groups, reducing agents and isosteres in drug discovery.^4^ Hence as these nuclei feature in many significant synthetic procedures their fast NMR detection would enable their analytical use as reaction probes and potentially the development of a role in pharmaceutical analysis. We exploit here the fact that molecular hydrogen exists in quantum states that span two manifolds, triplet or *ortho*hydrogen (*o*-H_2_) and singlet or *para*hydrogen (*p*-H_2_), to achieve this aim.

It was originally theorized by Bowers and Weitekamp that the spin order of *p*-H_2_ could be transformed into accessible nuclear spin polarisation through its chemical addition to a suitable molecular acceptor.^5,6^ The resulting non-Boltzmann spin state distribution creates what is now referred to as a hyperpolarised state which, when examined, gives rise to larger than normal NMR signals.^7^ In this work we use the signal amplification by reversible exchange (SABRE) variant of *p*-H_2_ induced polarisation, which achieves catalytic magnetisation transfer into a substrate.^8–10^ This effect occurs while the substrate is bound to a complex that also contains spin polarisation derived from *p*-H_2_ in the form of a pair of hydride ligands and the magnetisation is transferred through the *J*-coupling network into the nuclei of the ligand. The enhanced spin polarisation of the bound substrate is retained after dissociation and, when interrogated by NMR spectroscopy methods, it yields signals of substantially larger intensity than those which would normally be observed. In the case of pyridine, a ^1^H-signal enhancement factor of over 5500-fold has been reported at 9.4 T,^10^ and several reports on the performance of SABRE in biocompatible solvents such as ethanol and water have since been published.^11,12^ While a significant amount of attention has been dedicated to ^1^H SABRE NMR,^13–17^ this technique has also been shown to enable the hyperpolarisation of the heteronuclei ^13^C,^13,18–20^ ^15^N^21–27^ or ^31^P^28,29^ with the reported ^15^N signal gains being particularly noteworthy.^24^

In this report we use SABRE to hyperpolarise the ^119^Sn and ^29^Si nuclei of 5-(tributylstannyl)pyrimidine (**A**_Sn_) and 5-(trimethylsilyl)pyrimidine (**B**_Si_) respectively (Scheme 1). In order to achieve this result they are reacted with H_2_ and [IrCl(COD)(IMes)] (**1a**) or [IrCl(COD)(SIMes)] (**1b**) (where COD is 1,5-cyclooctadiene, IMes is 1,3-bis(2,4,6-trimethylphenyl)imidazol-2-ylidene and SIMes is 1,3-bis(2,4,6-trimethylphenyl)-4,5-dihydroimidazol-2-ylidene). This reaction forms the complexes [Ir(H)_2_(IMes)(**A**_Sn_)_3_]Cl (**2a**), [Ir(H)_2_(SIMes)(**A**_Sn_)_3_]Cl (**2b**), [Ir(H)_2_(IMes)(**B**_Si_)_3_]Cl (**3a**) and [Ir(H)_2_(SIMes)(**B**_Si_)_3_]Cl (**3b**) according to the pathway shown in Scheme 2 and they are SABRE active.

**Scheme 1:**
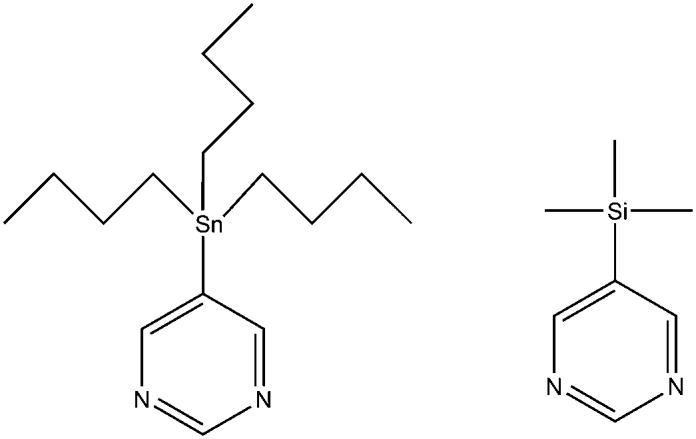
5-Tributylstannylpyrimidine (**A**_Sn_) and 5-trimethylsilylpyrimidine (**B**_Si_).

**Scheme 2:**
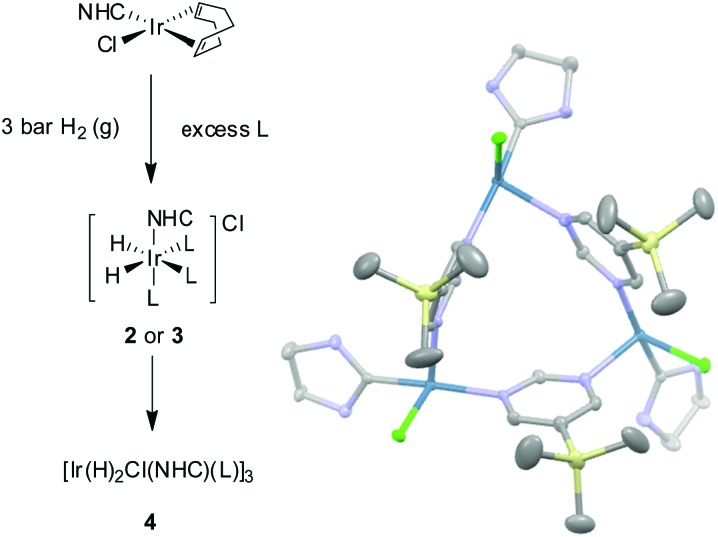
Reaction of [IrCl(COD)(NHC)] where NHC = IMes (**1a**) or SIMes (**1b**) with L (**A**_Sn_ or **B**_Si_) and H_2_ yields [Ir(H)_2_(IMes)(**A**_Sn_)_3_]Cl (**2a**), [Ir(H)_2_(IMes)(**B**_Si_)_3_]Cl (**3a**), [Ir(H)_2_(SIMes)(**A**_Sn_)_3_]Cl (**2b**) or [Ir(H)_2_(SIMes)(B)_3_]Cl (**3b**). In each case, a trinuclear complex of general formula [Ir(H)_2_Cl(NHC)(μ-pyrimidine-κ*N*:κ*N*′)]_3_ (**4**) eventually precipitates from these samples; the ORTEP for the product formed on reaction of **1b** with **B**_Si_ is shown to the right.

In the case of **2a**, the original orange solution turns pale yellow over a 5 minute period at 295 K and a hydride signal at *δ*_H_ –22.06 for [Ir(H)_2_(IMes)(**A**_Sn_)_3_]Cl (see ESI,‡ Table S27) appears in the corresponding ^1^H NMR spectrum. When such a sample is shaken with *p*-H_2_, SABRE is visible in the ^1^H resonances of free **A**_Sn_. By examining the role that the iridium catalysts concentration and the ligand excess play on the level of signal enhancement (see ESI‡) we found that a 2.5 mM concentration and 7-fold excess led to an 803 ± 73 fold increase in size of the H-2 signal, and a 1486 ± 156 fold increase for the combined H-4, H-6 signals of **A**_Sn_, respectively, after transfer at 70 G.

When SABRE transfer was repeated in a magnetic field of 25 G a ^119^Sn signal could be readily detected (Fig. 1). In this case, the ^119^Sn response receives its polarisation indirectly *via* the ^1^H polarisation of **A**_Sn_ rather than through a direct route as exemplified recently by Theis *et al.* for ^15^N^22^ and Koptyug for ^31^P.^29^ Fig. 2 shows how the resulting ^119^Sn signal enhancements and SNR values change with free ligand concentration when the concentration of **1a** is 5 mM. The 100 mM substrate loading, which corresponds to an excess of **A**_Sn_ to **2a** of 17 : 1, yielded the most intense ^119^Sn signal, with a SNR of 375 : 1 in the fully coupled spectrum, and 1099 : 1 in the decoupled spectrum. The signal enhancement obtained with this 100 mM loading was 772-fold (see ESI‡) and illustrates a time saving of in excess of 400 hours when set against the corresponding measurement under Boltzmann conditions.

**Fig. 1:**
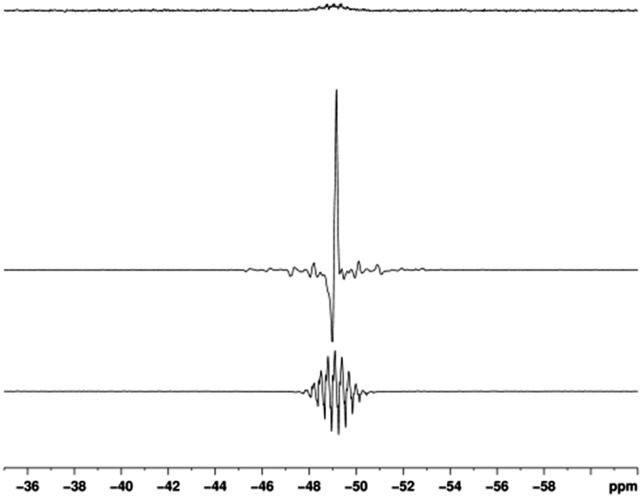
^119^Sn spectra of **A**_Sn_. Thermal trace acquired in Boltzmann equilibrium conditions using 3096 scans (top), hyperpolarised spectra acquired with (middle) and without butyl proton decoupling (bottom).

**Fig. 2:**
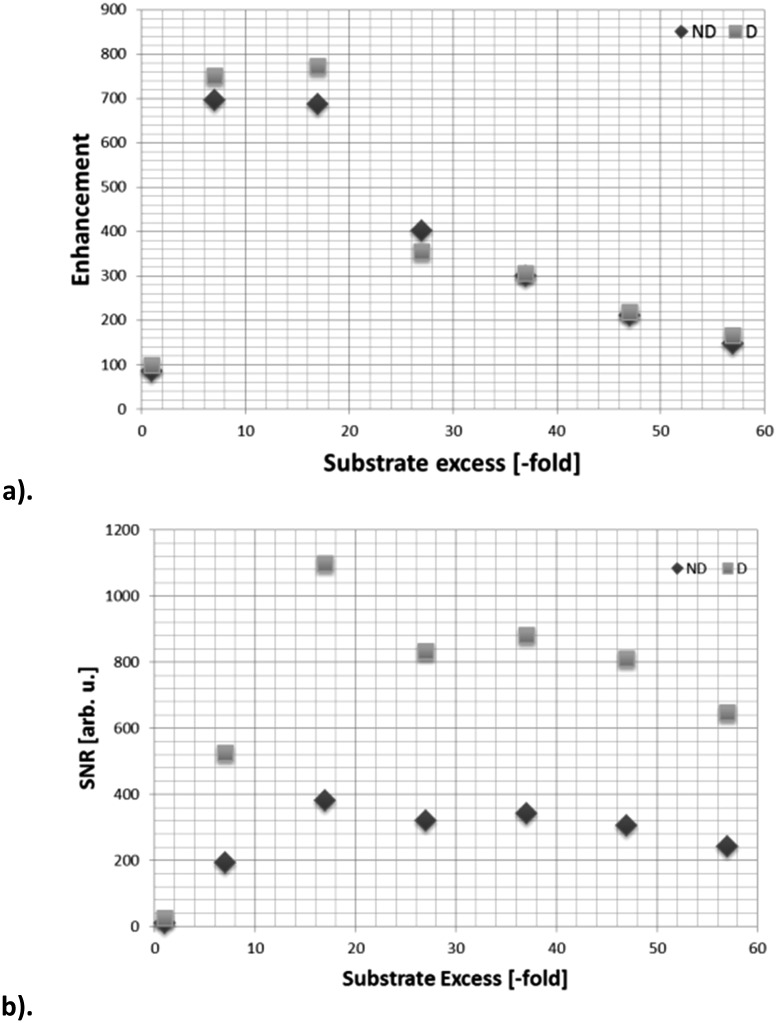
^119^Sn NMR data for the hyperpolarization of 5-(tributylstannyl)pyrimidine (**2**) in methanol-*d*_4_ through SABRE by **2a**; (a) signal enhancement as a function of ligand excess, relative to a 5 mM concentration of catalyst, (b) SNR as a function of ligand excess for the 5 mM loading. Data have been acquired with (D) and without (ND) butyl proton decoupling.

While the SIMes derived catalyst **1b** system forms the analogous SABRE active complex **2b** upon reaction with **A**_Sn_ it yields consistently lower enhancements than those seen with **2a** at 348 ± 47 and 301 ± 48 fold in the corresponding fully coupled and decoupled ^119^Sn NMR spectra. In order to rationalise this behaviour, the rates of H_2_ elimination from **2a** and **2b** were determined by exchange spectroscopy (EXSY) in methanol-*d*_4_ solution. At 295 K, H_2_ loss from **2a** proceeds at a rate of 0.56 s^–1^ whilst the analogous value for **2b** is 2.29 s^–1^. Furthermore, for **2a**, Δ*H* ^‡^ = 99.5 ± 3.4 kJ mol^–1^ and Δ*S* ^‡^ = 93.2 ± 11.6 J K^–1^ mol^–1^ while for **2b**, Δ*H* ^‡^ = 82.9 ± 2.9 kJ mol^–1^ and Δ*S* ^‡^ = 48.9 ± 10.1 J K^–1^ mol^–1^. These result in 
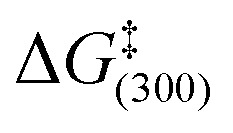
 values of 71.5 ± 0.1 kJ mol^–1^ and 68.3 ± 0.2 kJ mol^–1^ respectively for these transformations and confirm that **2b** undergoes more rapid ligand loss than **2a**, which for this substrate is detrimental to SABRE activity. It should be noted that these exchange rates are far lower than those that are optimal for transfer to pyridine^10,30,31^ which suggests the associated scalar coupling to a hydride ligand in **2** must be smaller in size.^32^

When the corresponding reaction between [IrCI(COD)(IMes)] (**1a**), 5-(trimethylsilyl)pyrimidine (**B**_Si_) and H_2_ in methanol-*d*_4_ is examined, the formation of **3a** is revealed. This complex yields a hydride signal at *δ*_H_ –22.44, which is almost identical in chemical shift to that of **2a**. A series of SABRE experiments were then performed which showed that the resulting ^1^H NMR signals of free **B**_Si_ were also strongly enhanced. When **1a** was used, transfer at 70 G ultimately yielded –761 ± 41 and –1524 ± 79 fold gains for the H-2 and the combined H-4, H-6 signals respectively. The corresponding values with **1b** were –450 ± 41 and –897 ± 82. Additionally, transfer to ^29^Si was observed and a non-decoupled SNR of 200 was obtained for a single shot measurement at a 25 mM concentration of substrate (Fig. 3).

**Fig. 3:**
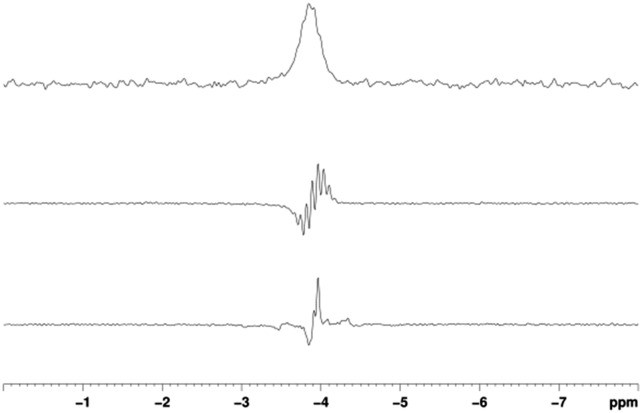
^29^Si spectra of a solution containing **B**_Si_. Thermal trace (top) acquired using 128 averages, hyperpolarised trace acquired using 1 average with (middle) and without (bottom) methyl protons decoupling.

The rates of H_2_ elimination from **3a** and **3b** were determined at 295 K and found to proceed at rates of 1.44 s^–1^ and 5.11 s^–1^ respectively. The corresponding activation parameters are Δ*H* ^‡^ = 104.7 ± 10.3 kJ mol^–1^ and Δ*S* ^‡^ = 119.9 ± 36.2 J K^–1^ mol^–1^ and Δ*H* ^‡^ = 90.4 ± 6.0 kJ mol^–1^ and Δ*S* ^‡^ = 82.3 ± 21.8 J K^–1^ mol^–1^ respectively. These result in 
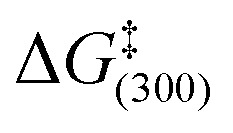
 values of 68.7 ± 0.1 kJ mol^–1^ and 65.7 ± 0.1 kJ mol^–1^ respectively and demonstrate again that the SIMes form undergoes more rapid ligand loss but this is detrimental to SABRE activity.

Interestingly, when these samples are left at 298 K the slow precipitation of a bright yellow powder is observed. X-ray diffraction studies on the analogous product formed with **B**_Si_ confirmed its identify as the iridium trimer **4** of general formula [Ir(H)_2_(Cl)(NHC)(μ-pyrimidine-κ*N*:κ*N*′)]_3_ (see ESI,‡ Sections 2.7 and 2.8). The formation of a related trinuclear iridium complex has been reported,^33^ alongside a series of related systems which feature bridging hydride ligands.^34,35^

In this paper we have described how the addition of H_2_ and the N-heterocycles 5-(tributylstannyl)pyrimidine (**A**_Sn_) and 5-(trimethylsilyl)pyrimidine (**B**_Si_) to [IrCl(COD)(IMes)] (**1a**) and [IrCl(COD)(SIMes)] (**1b**) result in the formation of a series of cationic dihydride complexes of the type [Ir(H)_2_(NHC)(L)_3_]Cl where L is **A**_Sn_ of **B**_Si_. These complexes prove to be able to catalyse the transfer of polarisation from *para*hydrogen into their NMR active groups thereby making them readily detectable. For example, the ^1^H NMR signal for the H-2 resonance of **A**_Sn_ shows an 827-fold intensity improvement when compared to that normally attained in Boltzmann equilibrium conditions. In the case of **B**_Sn_ the corresponding value is 761-fold.

When polarisation transfer to ^119^Sn is observed, a 700-fold intensity gain in its signal strength was measured which would require over 400 hours of measurement for comparable results. In contrast, the ^29^Si resonance of **B**_Si_ provided a 200-fold SNR gain in the analogous experiment. We believe such remarkable improvements could be exploited in the future for reaction monitoring in synthetic procedures such as those outline in the introduction.

The values of 
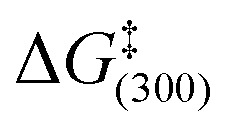
 for H_2_ loss in these complexes follow the order **2a** > **3a** > **2b** > **3b** and confirm that the remote silicon and tin centres of these pyrimidine substrates play a role in determining the ligand exchange rates even though they are isolated from the metal centre by four bonds. Furthermore, SABRE is a catalytic process, in which the product and starting materials chemical identity remains the same, but their magnetic properties are changed. Hence both catalyst deactivation and reaction selectivity reflect important points when analysing such processes, just like they would in a normal reaction. Here, the slow formation of a series of novel trimeric complexes with general formula [Ir(H)_2_(Cl)(NHC)(μ-pyrimidine-κ*N*:κ*N*′)]_3_ that have been characterised by X-ray crystallography for **B**_Si_ results in the need to employ fresh samples during the SABRE analysis. Hence while high levels of signal gain are readily achieved through SABRE, there is a need to refresh the catalyst periodically if activity is to be maintained over several hours.

The EPSRC (grant no. EP/G009546/1) and the Wellcome Trust (092506 and 098335) are thanked for funding. We also acknowledge Bruker Biospin for support and help from James Hayes.
