# Supplementary material for: Using signal amplification by reversible exchange (SABRE) to hyperpolarise 119Sn and 29Si NMR nuclei
Source: Chem Commun (Camb). 2016 Nov 23;52(100):14482–5. doi: 10.1039/c6cc07109k (PMC5436037; doi:10.1039/c6cc07109k)
Supplement: Supplementary file 1 [file CC-052-C6CC07109K-s001.pdf]

Electronic Supplementary Material (ESI) for ChemComm.
This journal is © The Royal Society of Chemistry 2016

Using Signal Amplification by Reversible Exchange (SABRE) to hyperpolarise ^{119}Sn and ^{29}Si NMR nuclei

Alexandra M. Olaru,[a] Alister Burt,[a] Peter J. Rayner,[a] Sam Hart,[a] Adrian C. Whitwood,[a] Gary G. R. Green[a] and Simon B. Duckett*[a]

1. Materials and methods

1.1 Materials

1.2 Synthesis of 5-(trimethylsilyl)pyrimidine

1.3 Instrumentation and procedures

2. Experimental Results

2.1 ^{1}H SABRE polarisation of 2a

2.2 ^{119}Sn SABRE polarisation of 2a and 2b

2.3 Rates of H_2 loss for 2a and 2b

2.4 ^{1}H SABRE polarisation of 3a and 3b

2.5 ^{29}Si SABRE polarisation of 3a and 3b

2.6 Rates of H_2 loss for 3a and 3b

2.7 Complex characterisation by XRD – 3a

2.8 Complex characterisation by XRD – 3b

2.9 Complex characterisation by NMR

3. References

1. Materials and Methods

1.1 Materials

All the experimental procedures that are associated with this work were carried out under nitrogen using standard Schlenk techniques. The solvents used in the synthetic chemistry were dried using an Innovative Technology anhydrous solvent system, or distilled from an appropriate drying agent under nitrogen. Catalysts were prepared according to literature methods [1].

Deuterated methanol and tributylstannyl pyrimidine were obtained from Sigma-Aldrich and used as supplied. 5-(trimethylsilane)pyrimidine was synthesised as described below.

1.2 Synthesis of 5-(trimethylsilyl)pyrimidine

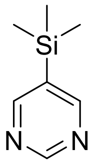

Magnesium (1.2 g, 50 mmol, 1.25 eq.) was added to a stirred solution of trimethylsilylchloride (5.43 g 50 mmol, 1.25 eq.) in 1,3-dimethyl-2-imidazolidinone (DMI) (50 mL) at rt under N_2(g). The resulting suspension was heated at 80 °C for 4 h. Then, a solution of 5-bromopyrimidine (6.40 g, 40 mmol, 1.0 eq.) in DMI (50 mL) was added whilst maintaining the temperature at 80 °C. The resulting solution was heated at 75 °C for 18 h and then cooled to room temperature. Water (200 mL) was added and the solution was adjusted to pH 10 with 2 M $NaOH_{(aq)}$. The solution was extracted with EtOAc (3 x 250 mL) and the combined organic layers were washed with water (4 x 250 mL), dried ($MgSO_4$) and concentrated under reduced pressure to give the crude product. Purification by flash column chromatography on silica with 9:1-8:2 hexane-EtOAc as eluent gave 5-(trimethylsilyl)pyrimidine (341 mg, 5.6%) as a colourless oil, R_F (8:2 hexane-EtOAc) 0.2; ^{1}H NMR (400 MHz, $CDCl_3$) δ 9.06 (s, 1H), 8.65 (s, 2H), 0.22 (s, 9H); ^{13}C NMR (100.6 MHz, $CDCl_3$) δ 161.0, 158.8, 132.2, 1.71; MS (ESI) m/z 153 [$(M + H)^+$, 100]; HRMS m/z calculated for $C_7H_{12}N_2Si$ $(M + H)^+$ 153.0843, found 153.0837 (+3.8 ppm error).

1.3 Instrumentation and procedures

All NMR measurements were recorded on Bruker Avance III series 400 MHz or 500 MHz systems. NMR samples were prepared in 5 mm NMR tubes fitted with Young's valves. Samples were degassed prior to *p*-H_2 (3 bars) addition. NMR characterization data was collected using a range of 1 and 2D methods that included nOe, COSY and HMQC procedures [2-6]. The slow dynamic processes exhibited by the complexes studied here were monitored by EXSY methods [7]. The quoted ^{119}Sn chemical shifts are relative to that of $SnMe_4$ being zero at an absolute frequency of 32.290655 MHz when ^{1}H is 100.00 MHz.

SABRE analysis NMR samples were prepared containing **2** and **3** in 0.6 ml of methanol-d_4. Arrays of NMR measurements were collected using various concentrations of the complexes formed with **1a** and **1b** (**a** denotes the complex formed with the IMes catalyst, while **b** stands for the SIMes catalyst, as described in the results section of the main text). After adding p-H_2 at 3 bar pressure ^1H NMR spectra were recorded using π/2 excitation pulses immediately after shaking the sample in a magnetic field of 65 G. ^{29}Si and ^{119}Sn were hyperpolarised in a similar fashion, but using a PTF of approximately 25 G. Enhancement factors were calculated by using the ratio of the integral areas of individual resonances in the hyperpolarised spectra and the spectrum collected under normal H_2 and Boltzmann equilibrium conditions respectively. Antiphase spectra were processed in magnitude mode.

EXSY measurements and kinetic analysis A series of exchange spectroscopy (EXSY) measurements were made to probe the dynamic behaviour of these systems. This process involved the selective excitation of a single resonance and the subsequent measurement of a ^1H NMR spectrum at time, t, after the initial pulse. The resulting measurements consisted of a series of data arrays such that t was varied over 10-25 values, typically from 0.1 to 1.0 s, to encode the reaction profile. The precise values were varied with temperature to suit the speed of the process. Data was collected for a range of temperatures and sample concentrations.

Integrals for the interchanging peaks in the associated ^1H EXSY spectra were obtained and converted into a percentage of the total detected signal. These data were then analysed as a function of the mixing time according to a differential kinetic model [8]. Rates of exchange were determined by employing a Runge-Kutta [9, 10] scheme to solve the system of differential equations and a Levenberg–Marquardt algorithm [11, 12] to minimise the sum of residuals in the associated nonlinear least squares analysis. The theoretical model used to fit the experimental EXSY data involved a two-site exchange (A ↔ B), as expressed by the equations below:

$$-\frac{dA}{dt} = -K_{ab} * A + K_{ba} * B$$

$$-\frac{dB}{dt} = + K_{ab} * A - K_{ba} * B$$

An example of typical build-up/decay curves obtained from the integration of the experimental EXSY data, together with the corresponding fitted data is presented in Figure S1.

Thermodynamic parameters were calculated using the exchange rates obtained for a wide range of temperatures (detailed below) and the Eyring-Polanyi equation [13, 14].

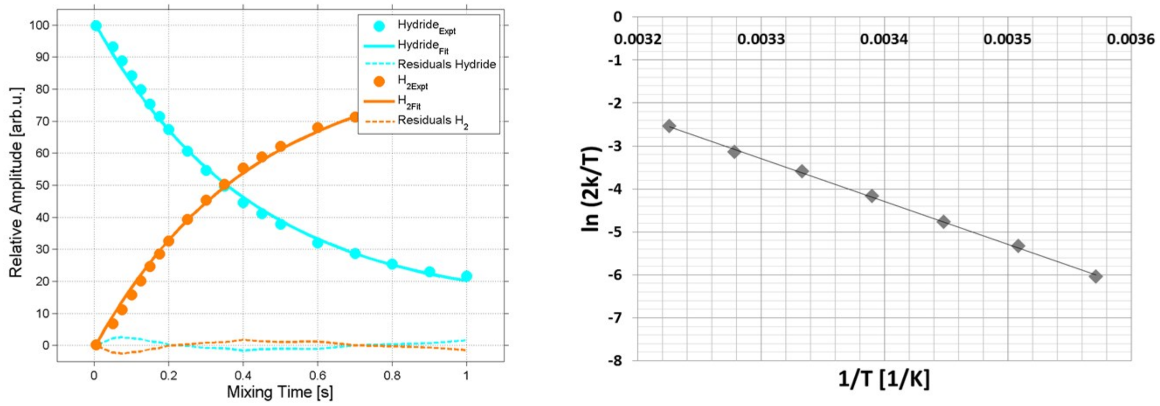

Figure S1: Build-up/decay obtained from the integration of experimental EXSY data recorded for 2b at 295 K (left) and corresponding Eyring plot for the H_2 loss process (right).

2. Experimental results

2.1 ^{1}H SABRE polarisation of 2a.

We have examined the effect of concentration and ligand excess on the level of proton signal enhancement that is detected under SABRE conditions by preparing sets of samples in which:

i). The concentration of **1a** was kept at 5 mM while the excess of **2** was progressively increased.

ii). The ratio between **1a** and **2** was kept constant while the concentration **1a** was progressively increased.

For each sample, typically 5 to 7 hyperpolarised spectra were acquired and the integral areas of each peak were averaged and divided by the corresponding integral obtained from a spectrum acquired in Boltzmann equilibrium conditions. Errors for the resulting individual enhancements have been calculated using the standard deviation formula, taking into account the limited number of population samples. The errors for the total enhancements have been determined using the formula for the Gaussian propagation of errors.

In order to determine the effect of the substrate loading, a series of samples containing 5 mM of **1a** and increasing excess of **2** were prepared in methanol-d_4 and examined for SABRE polarisation. The results and the associated errors are detailed in Table S1 and depicted in Figure S2. It can be seen that the signal enhancement for both sites decreases with increasing ligand excess, from -2036 (1-fold excess) to -345 (27-fold excess), in a similar way to other substrates previously examined [15].

Table S1. ^{1}H SABRE enhancement as a function of substrate loading for 2a.

Excess [-fold]	ε (H-2) [arb. u.]	ε (H-4, H-6) [arb. u.]	ε (total) [arb. u.]
1	-827 ± 86	-1210 ± 130	-2036 ± 156
4	-551 ± 55	-973 ± 99	-1524 ± 113
7	-538 ± 42	-1003 ± 83	-1541 ± 93
12	-411 ± 26	-751 ± 42	-1162 ± 49
17	-282 ± 26	-477 ± 96	-759 ± 99
27	-143 ± 14	-203 ± 18	-345 ± 23

It must be noted that usually, as the efficiency of the polarisation transfer process directly depends on the scalar coupling between the ligand's protons and the hydrides, each resonance will be differently enhanced, as a function of the proton's relative position to the binding site. The complexity of this process is furthermore increased by the fact that the individual protons also have different relaxation times, so the enhancement obtained will be affected by the rate of signal decay. In the case of **2** all three resonances in the pyrimidine ring are *ortho* to a nitrogen atom, so their enhancements are comparable, the value for H-2 being slightly higher than half of the one obtained for the sum of H-4 and H-6 (for which individual enhancements could not be determined due to peak overlap).

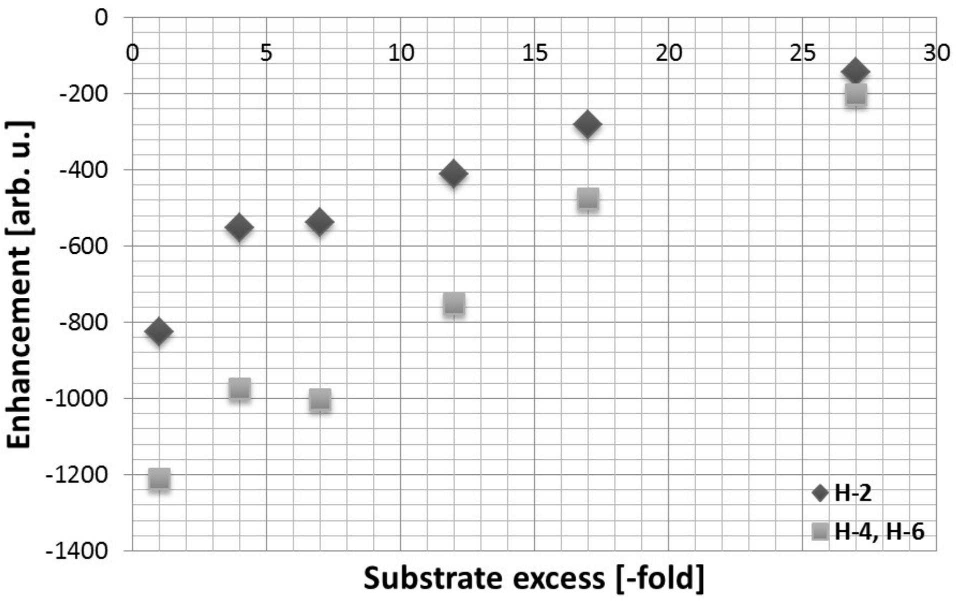

Figure S2. ^{1}H SABRE enhancement as a function of substrate loading for 2a.

In order to establish the effect the catalyst loading has on the enhancements, samples containing **1a** and **2** in a 1:10 ratio (7-fold excess) have been prepared by dilution from a stock solution of 12.5 mM concentration and were examined for SABRE polarisation. (While the complex prepared with just 1-fold excess gave better enhancement values, the optimal ratio was chosen to be 1:10 due to considerably higher SNR values). The results and the associated errors are detailed in Table S2 and pictured in Figure S3.

Table S2. ^{1}H SABRE enhancement as a function of catalyst concentration for 2a.

Catalyst concentration [mM*dm^{-3}]	ε (H-2) [arb. u.]	ε (H-4, H-6) [arb. u.]	ε (total) [arb. u.]
2.5	-803 ± 73	-1486 ± 156	-2289 ± 179
5.0	-505 ± 57	-962 ± 101	-1467 ± 116
7.5	-406 ± 89	-795 ± 174	-1201 ± 196
10.0	-305 ± 24	-548 ± 20	-853 ± 31
12.5	-331 ± 29	-631 ± 62	-962 ± 69

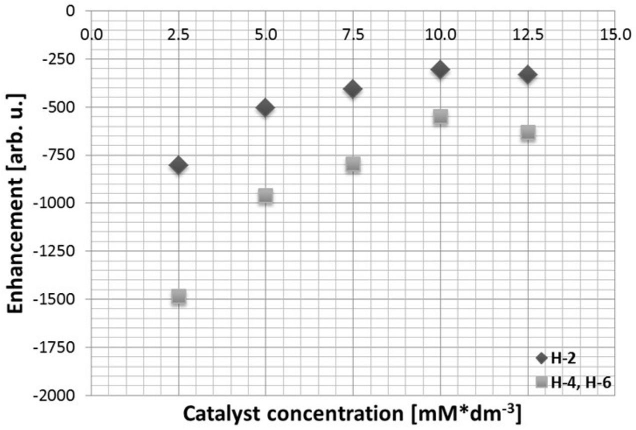

Figure S3. ^{1}H SABRE enhancement as a function of substrate concentration for 2a.

After optimising the substrate loading and the complex concentration, the maximum ^{1}H SABRE enhancement obtained was -2289 (see figures S2 and S3).

2.2 ^{119}Sn SABRE polarisation of 2a and 2b.

In order to detect a ^{119}Sn signal, a series of shake and drop experiments were performed after 5 seconds of polarisation transfer in a magnetic field of ~25 G on samples containing **2a and 2b**. ^{119}Sn SABRE hyperpolarisation was indeed observed (Figure S4). Due to the SNR values for **2a** (380 and 1098 for not-decoupled and decoupled spectra respectively) being considerably higher than for **2b** (185 and 432 for not-decoupled and decoupled spectra respectively), **2a** was chosen for a series of optimisation experiments that were conducted to determine the sample composition that leads to the best signal gain.

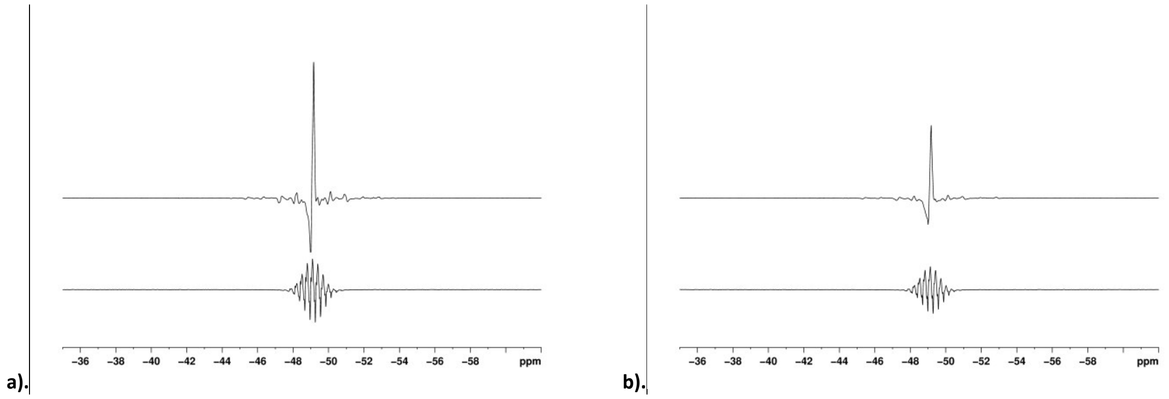

Figure S4. ^{119}Sn hyperpolarised spectra without ^{1}H decoupling (bottom) and with ^{1}H decoupling applied on the butyl protons (top) of a). 2a and b). 2b.

First, samples were prepared at various ratios of catalyst to substrate, with a set catalyst concentration of 5 mmol*dm^{-3}. SNR values were calculated for spectra acquired without ^{1}H decoupling (ND) and with ^{1}H decoupling pulses applied on the butyl resonances. Figures S4 and S5 show the resulting plots of ^{119}Sn signal enhancement, and SNR values, as a function of the free ligand concentration.

Table S3. ^{119}Sn SNR as a function of substrate loading/excess for 2a.

Substrate loading [mM*dm^{-3}]	Excess [-fold]	SNR (ND)	SNR (D)
20	1	9 ± 1	28 ± 5
50	7	190 ± 22	534 ± 146
100	17	375 ± 130	1099 ± 93
150	27	328 ± 14	758 ± 75
200	37	326 ± 13	868 ± 93
250	47	287 ± 21	773 ± 49
300	57	239 ± 18	705 ± 67

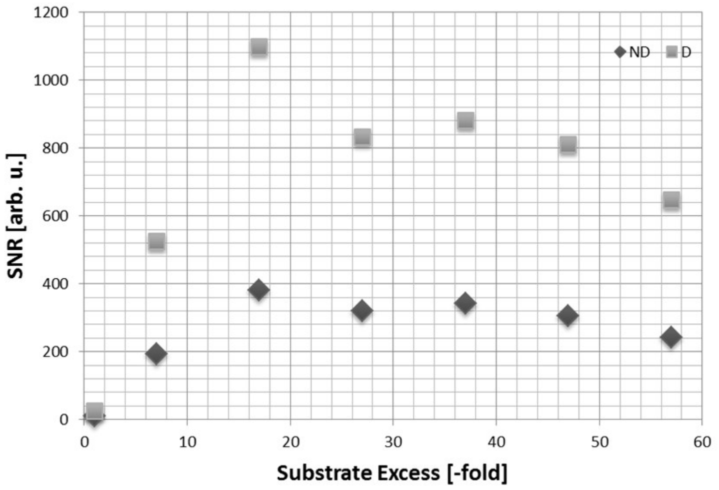

Figure S4. ^{119}Sn SNR as a function of substrate loading for 2a.

The enhancement values for ^{119}Sn have been calculated by dividng the average SNR of the hyperpolarised spectra to the SNR of the spectrum of a reference sample, prepared with 5 mM **1a** and 100 mM **2**, acquired in Boltzmann equilibrium conditions using 3096 scans.

Table S4. ^{119}**Sn enhancement as a function of substrate loading for 2a.**

Substrate loading [mM*dm^{-3}]	Excess [-fold]	ε (ND) [arb. u.]	ε (D) [arb. u.]
20	1	86	98
50	7	696	750
100	17	687	772
150	27	400	355
200	37	299	305
250	47	211	217
300	57	146	165

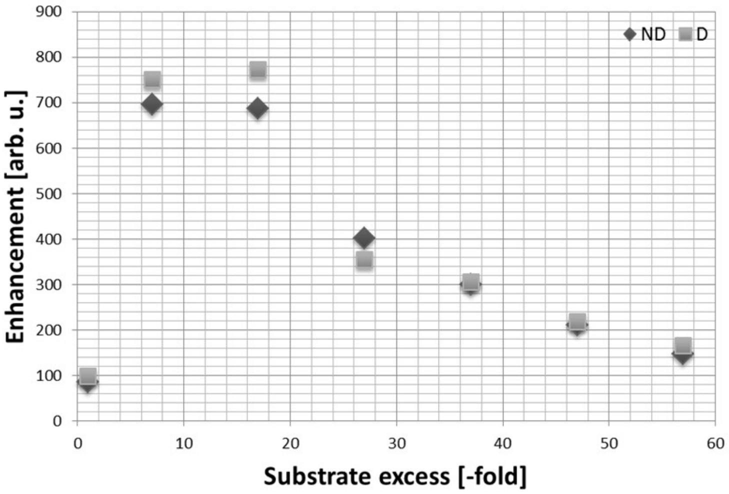

Figure S5. ^{119}**Sn enhancement as a function of substrate loading for 2a.**

The 100 mM substrate loading proved to yield the most intense ^{119}Sn signal. Hence a catalyst to substrate ratio of 1:20 (17-fold excess of substrate to catalyst) was employed in further series of optimisation measurements. The results are presented in tables T-S5 and T-S6 and depicted in figures S6 and S7.

Table S5. ^{119}**Sn SNR as a function of catalyst loading for 2a.**

Catalyst concentration [mM*dm^{-3}]	SNR (ND) [arb. u.]	SNR (D) [arb. u.]
0.5	22 ± 2	70 ± 2
2.5	153 ± 4	398 ± 7
5.0	118 ± 8	544 ± 30
7.5	289 ± 2	749 ± 31
10.0	274 ± 11	619 ± 51
12.5	274 ± 4	582 ± 50
15.0	181 ± 7	439 ± 14

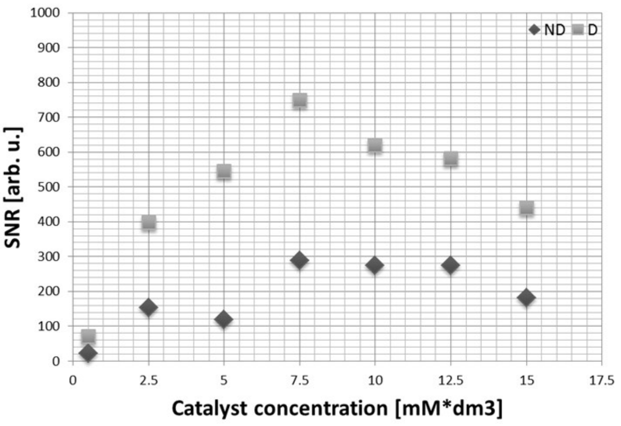

Figure S6. **^{119}Sn SNR as a function of catalyst concentration for 2a.**

Table S6. **^{119}Sn enhancement as a function of catalyst concentration for 2a.**

Catalyst concentration [mM*dm^{-3}]	ε (ND) [arb. u.]	ε (D) [arb. u.]
0.5	405	491
2.5	574	556
5.0	222	380
7.5	362	349
10.0	258	216
12.5	207	163
15.0	114	102

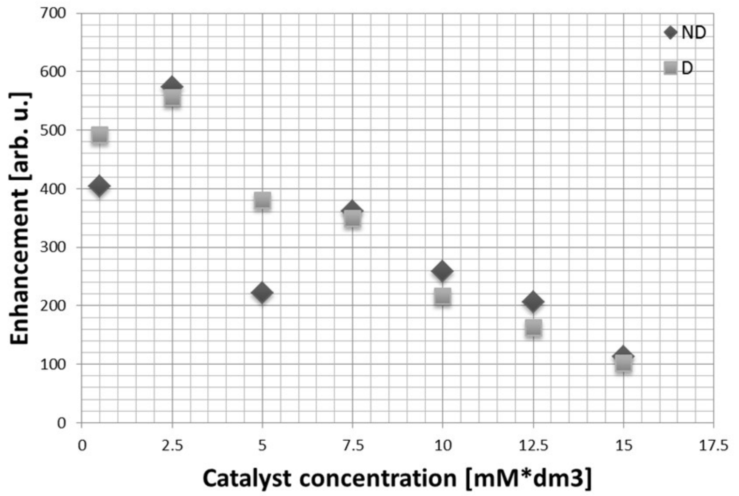

Figure S7. **^{119}Sn enhancement as a function of catalyst concentration for 2a.**

2.3 Rates of H_2 loss for 2a and 2b.

The rates of H_2 elimination from complexes **2a** and **2b** have been determined by EXSY spectroscopy over the temperature range 280-315 K and 275-310 K respectively in methanol-d_4 solution. The results are presented below, in tables Table S7 and Table S8 (corresponding activation parameters).

Table S7. H_2 loss rates for 2a and 2b as a function of temperature for 7.5 mM catalyst and 17-fold substrate excess.

	**2a**	**2b**
Temperature [K]	*k* [1/s]	*k* [1/s]
270	-	0.03 ± 0.01
275	-	0.11 ± 0.01
280	0.06 ± 0.01	0.33 ± 0.01
285	0.13 ± 0.01	0.69 ± 0.01
290	0.25 ± 0.01	1.23 ± 0.01
295	0.57 ± 0.01	2.29 ± 0.02
300	1.14 ± 0.02	4.11 ± 0.03
305	2.23 ± 0.02	6.59 ± 0.10
310	4.12 ± 0.13	12.20 ± 0.18
315	6.98 ± 0.22	-

Table S8. Activation parameters for 2a and 2b.

**Activation Parameters**	**2a**	**2b**
$\Delta H^{\neq}$ (kJ mol^{-1})	100	83
+/-	3	3
$\Delta S^{\neq}$ (J K^{-1} mol^{-1})	93	49
+/-	12	10
$\Delta G^{\neq}_{300}$ (kJ mol^{-1})	71.5	68.3
+/-	0.1	0.2
R Square	0.998	0.999

2.4 ^{1}H SABRE hyperpolarisation of 3a and 3b.

Samples containing **3a** and **3b** were prepared, using the optimum complex concentrations and number of ligand equivalents determined for **2a** and **2b**, namely 2.5 mM (1:10, 7-fold excess of substrate) for ^{1}H and 7.5 mM (1:20, 17-fold excess of substrate) for ^{29}Si. ^{1}H SABRE enhanced spectra of **3a** and **3b** are presented below, in Figure S8.

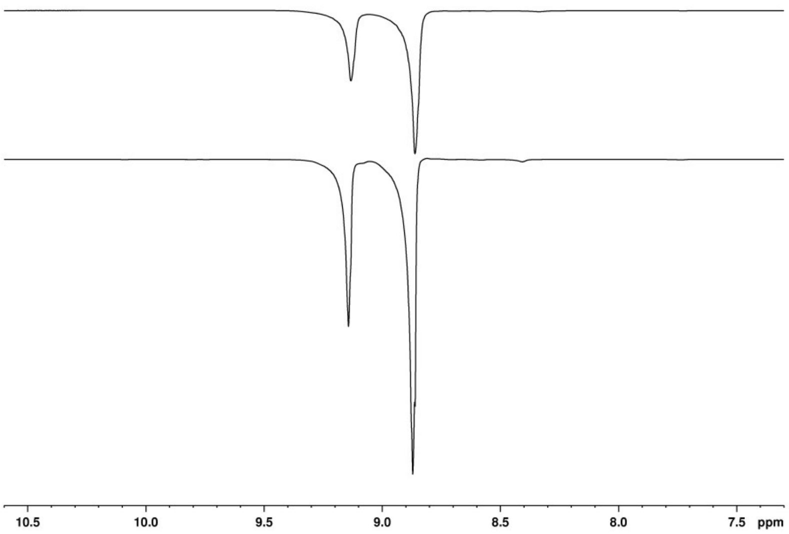

Figure S8. ^1H hyperpolarised spectra of 3a (bottom) and 3b (top). Samples have been prepared using the amounts optimised for 2a/2b: 2.5 mM of 1a and b respectively and 25 mM of 3.

The enhancement values were calculated as described previously and detailed in Table S9.

Table S9. ^1H enhancements for 3a and 3b.

	**ε (H-2) [arb. u.]**	**ε (H-4, H-6) [arb. u.]**	**ε (total) [arb. u.]**
**3a**	-761 ± 41	-1524 ± 79	-2285 ± 89
**3b**	-450 ± 41	-897 ± 82	-1347 ± 92

2.5 ^{29}Si SABRE hyperpolarisation of 3a and 3b.

SABRE hyperpolarisation experiments have been performed for ^{29}Si at 25 G. ^{29}Si hyperpolarised spectra, acquired both with, and without, methyl proton decoupling, are presented in Figure S9, together with a thermal trace acquired using 128 scans. The single shot ^{29}Si hyperpolarised spectrum has an SNR of 71, while the thermal trace SNR is 15 (which corresponds to an SNR value of 1.4 per scan). The acquisition time for the spectrum using magnetisation at thermal equilibrium was 21 h 20 min.

Table S10. SNR values obtained for 3a and 3b as a function of concentration and ligand loading obtained from experiments without (ND) and with (D) ^1H decoupling of the methyl protons.

**Sample**	**SNR [arb.u] ND**	**SNR [arb.u] D**
**3a** (2.5 mM **1a**, 7-fold substrate excess)	200 ± 14	27 ± 5
**3b** (2.5 mM **1b**, 7-fold substrate excess)	81 ± 3	9 ± 1
**3a** (7.5 mM **1a**, 17-fold substrate excess)	118 ± 20	13 ± 1
**3b** (7.5 mM **1b**, 17-fold substrate excess)	95 ± 10	19 ± 2

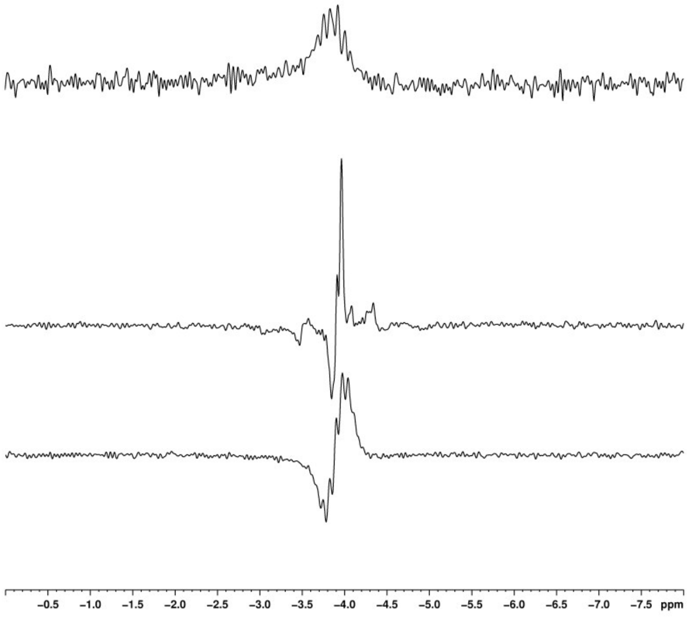

Figure S9. ^{29}Si thermal trace for 3a (top) acquired using 128 averages and hyperpolarised spectra (bottom).

2.6 Rates of H_2 loss for 3a and 3b

The rates characterising the H_2 loss process were measured as described above and are listed in Table S11.

Table S11. H_2 loss rates for 3a and 3b as a function of concentration at 295 K.

Complex	H_2 loss rate [1/s]
**3a** (2.5 mM **1a**, 7-fold substrate excess)	6.06 ± 0.14
**3b** (2.5 mM **1b**, 7-fold substrate excess)	18.40 ± 1.14
**3a** (7.5 mM **1a**, 17-fold substrate excess)	1.44 ± 0.02
**3b** (7.5 mM **1b**, 17-fold substrate excess)	5.11 ± 0.07

As for the ^{119}Sn compounds, the rates of H_2 loss are considerably higher for **3b** when compared to **3a**. It is also worth noting that for the compounds with a concentration and catalyst:substrate ratio optimised for ^{1}H polarisation are 3 to 6 times higher than for the complexes optimised for ^{119}Sn polarisation.

Table S12. H_2 loss rates for 3a and 3b as a function of temperature for 7.5 mM catalyst and 17-fold substrate excess.

**Complex**	**3a**	**3b**
Temperature [K]	*k* [1/s]	*k* [1/s]
260	-	0.03 ± 0.01
265	-	0.08 ± 0.01
270	-	0.19 ± 0.01
275	0.06 ± 0.01	0.43 ± 0.01
280	0.16 ± 0.01	0.86 ± 0.01
285	0.40 ± 0.01	1.58 ± 0.11
290	0.82 ± 0.01	2.89 ± 0.03
295	1.44 ± 0.02	5.11 ± 0.07
300	3.33 ± 0.03	-

Table S13. Activation parameters for 3a and 3b.

**Activation Parameters**	**3a**	**3b**
$\Delta H^{\neq}$ (kJ mol^{-1})	104.7	90.4
+/-	10.3	6.0
$\Delta S^{\neq}$ (J K^{-1} mol^{-1})	119.9	82.3
+/-	36.2	21.8
$\Delta G^{\neq}_{300}$ (kJ mol^{-1})	68.7	65.7
+/-	0.1	0.1
R Square	0.994	0.995

2.7 Complex characterisation by XRD – 3a

X-ray diffraction of the tributyl tin analogue was attempted. The crystals proved to be of too poor quality (even with synchrotron radiation) to produce a complete structure due to poor mosaicity and considerable disorder of the butyl side chains. However, it was apparent that the complex had the same cyclic trimer structure as the silicon-containing complexes now described.

2.7.1 Experimental

Single crystals of $C_{84}H_{114}Cl_3Ir_3N_{12}Si_3$ were prepared in methanol-*d*$_4$, under 3 bar H_2. A suitable crystal was selected, immersed in oil and placed on a micromount on a SuperNova, Dual, Cu at zero, Eos diffractometer. The crystal was kept at 110.05(10) K during data collection. Using Olex2 [16], the structure was solved with the ShelXS [17] structure solution program using Direct Methods and refined with the ShelXL [18] refinement package using Least Squares minimisation.

2.7.2 Crystal structure determination of $C_{84}H_{114}Cl_3Ir_3N_{12}Si_3$

Crystal Data for C84H114Cl3Ir3N12Si3 (M =2059.09 g/mol): monoclinic, space group P21/c (no. 14), a = 20.9974(5) Å, b = 14.9781(4) Å, c = 31.1032(9) Å, β = 94.152(3)°, V = 9756.4(4) Å3, Z = 4, T = 110.05(10) K, μ(CuKα) = 9.206 mm-1, Dcalc = 1.402 g/cm3, 33778 reflections measured (7.334° ≤ 2Θ ≤ 134.16°), 17287 unique (Rint = 0.0319, Rsigma = 0.0438) which were used in all calculations. The final R1 was 0.0411 (I > 2σ(I)) and wR2 was 0.1026 (all data).

2.7.3 Refinement model description

Number of restraints - 0, number of constraints - unknown.

Details:

1. Fixed Uiso

At 1.2 times of: All C(H) groups.

At 1.5 times of: All C(H,H,H) groups, All Ir(H,H) groups.

2.a. Riding coordinates: Ir1(H,HA), Ir2(HB,HC), Ir3(HD,HE).

2.b. Aromatic/amide H refined with riding coordinates:

C2(H2), C3(H3), C6(H6), C8(H8), C15(H15), C17(H17), C22(H22), C23(H23), C25(H25), C30(H30), C31(H31), C34(H34), C36(H36), C43(H43), C45(H45), C50(H50), C51(H51), C53(H53), C58(H58), C59(H59), C62(H62), C64(H64), C71(H71), C73(H73), C78(H78), C79(H79), C81(H81).

2.c. Idealised Me refined as rotating group:

C10(H10A,H10B,H10C), C11(H11A,H11B,H11C), C12(H12A,H12B,H12C), C19(H19A,H19B,H19C), C20(H20A,H20B,H20C), C21(H21A,H21B,H21C), C26(H26A,H26B,H26C), C27(H27A,H27B,H27C), C28(H28A,H28B,H28C), C38(H38A,H38B,H38C), C39(H39A,H39B,H39C), C40(H40A,H40B,H40C), C47(H47A,H47B,H47C), C48(H48A,H48B,H48C), C49(H49A,H49B,H49C), C54(H54A,H54B,H54C), C55(H55A,H55B,H55C), C56(H56A,H56B,H56C), C66(H66A,H66B,H66C), C67(H67A,H67B,H67C), C68(H68A,H68B,H68C), C75(H75A,H75B,H75C), C76(H76A,H76B,H76C), C77(H77A,H77B,H77C), C82(H82A,H82B,H82C), C83(H83A,H83B, H83C), C84(H84A,H84B,H84C).

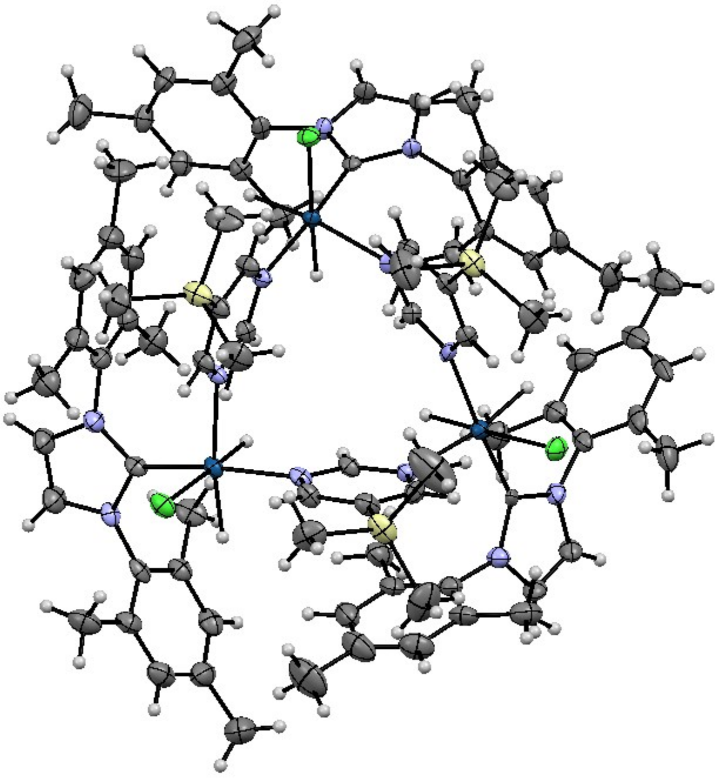

Figure S10. XRD structure of $C_{84}H_{114}Cl_3Ir_3N_{12}Si_3$.

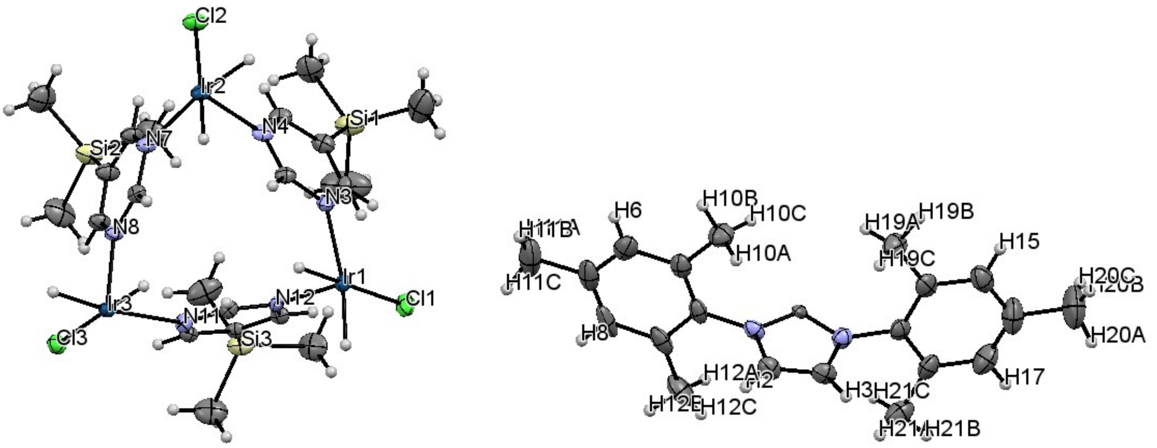

a). **b).**

Figure S11. Selected aspects of the XRD structure of $C_{84}H_{114}Cl_3Ir_3N_{12}Si_3$: a). Representation of central trimer without the three carbene units attached to the Ir atoms; b). Representation of the carbine unit with the corresponding atom labels.

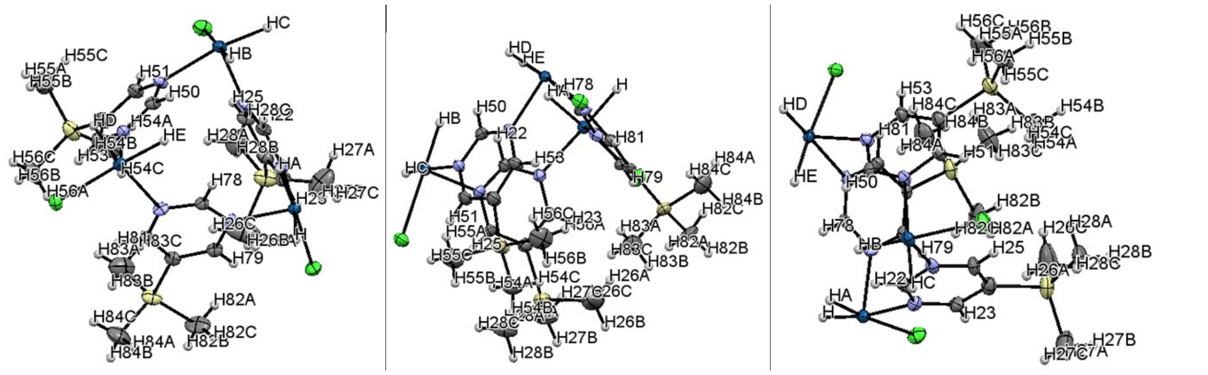

Figure S12. Selected aspects of the XRD structure of $C_{84}H_{114}Cl_3Ir_3N_{12}Si_3$: Representation of central trimer without the three carbene units attached to the Ir atoms and the corresponding labels for the H atoms. For clarity the trimer is presented from three different perspectives.

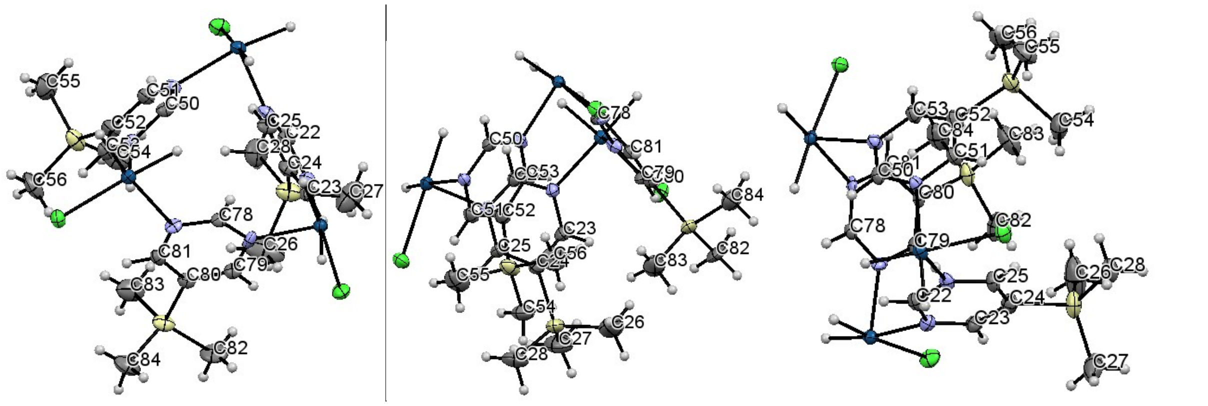

Figure S13. Selected aspects of the XRD structure of $C_{84}H_{114}Cl_3Ir_3N_{12}Si_3$: Representation of central trimer without the three carbene units attached to the Ir atoms and the corresponding labels for the C atoms. For clarity the trimer is presented from three different perspectives.

Table S14. Fractional Atomic Coordinates (×10[4]) and Equivalent Isotropic Displacement Parameters (Å[2]×10[3]) for $C_{84}H_{114}Cl_3Ir_3N_{12}Si_3$. U_{eq} is defined as 1/3 of of the trace of the orthogonalised U_{IJ} tensor.

Atom	*x*	*y*	*z*	U(eq)
C1	2113(3)	4274(4)	3985.3(16)	18.3(11)
C2	1659(3)	5650(4)	4043.5(19)	30.6(14)
C3	2131(3)	5730(4)	3782.0(19)	29.9(14)
C4	1143(3)	4425(4)	4415.2(19)	28.9(14)
C5	576(3)	4118(4)	4208(2)	31.9(14)
C6	86(3)	3806(4)	4454(2)	40.5(17)
C7	158(4)	3816(5)	4898(2)	43.4(19)
C8	720(4)	4155(5)	5093(2)	44(2)
C9	1225(4)	4465(4)	4864(2)	37.1(16)
C10	493(3)	4097(5)	3722(2)	38.7(16)
C11	-348(5)	3484(6)	5170(3)	68(3)
C12	1826(4)	4844(6)	5073(2)	50(2)
C13	2877(3)	4731(4)	3446.1(19)	28.0(13)
C14	2688(3)	4428(4)	3031(2)	34.8(15)
C15	3157(4)	4302(5)	2749(2)	42.5(17)
C16	3796(4)	4505(6)	2861(3)	55(2)
C17	3959(3)	4812(5)	3271(3)	46.9(18)
C18	3510(3)	4924(5)	3576(2)	35.8(15)
C19	2002(3)	4237(5)	2897(2)	40.3(17)
C20	4292(5)	4359(8)	2540(3)	78(3)
C21	3706(4)	5217(5)	4027(2)	44.1(17)
C22	2909(3)	2335(4)	3269.4(17)	23.0(12)
C23	3693(3)	2752(4)	3780.8(18)	25.6(12)
C24	4169(3)	2370(4)	3551.4(19)	28.1(13)
C25	3945(3)	1957(4)	3158.9(18)	24.6(12)
C26	5049(4)	1890(9)	4318(3)	89(4)
C27	5289(4)	3571(6)	3818(3)	65(3)
C28	5513(3)	1750(5)	3403(3)	47.3(19)
C29	2618(3)	1031(4)	1839.1(17)	22.4(12)
C30	2330(3)	1090(5)	1118(2)	37.5(16)
C31	2205(3)	259(5)	1244.6(19)	36.2(15)
C32	2650(3)	2515(4)	1473.9(16)	25.1(13)
C33	2148(3)	3028(4)	1606.5(18)	27.2(13)
C34	2210(3)	3946(5)	1615(2)	34.1(15)
C35	2772(4)	4352(5)	1504(2)	39.0(16)
C36	3256(3)	3829(5)	1359(2)	37.9(16)
C37	3202(3)	2901(4)	1340(2)	32.6(14)
C38	1551(3)	2587(5)	1748(2)	34.7(15)
C39	2847(5)	5362(5)	1547(3)	57(2)
C40	3737(3)	2330(5)	1194(2)	42.8(17)
C41	2246(3)	-546(4)	1936.2(19)	29.9(13)
C42	1653(3)	-627(4)	2110.8(19)	32.3(14)
C43	1535(4)	-1397(5)	2344(2)	38.1(16)
C44	1992(4)	-2069(5)	2403(2)	42.2(17)
C45	2564(4)	-1976(5)	2213(2)	41.1(16)
C46	2712(3)	-1219(4)	1977(2)	35.5(15)
C47	1162(3)	94(5)	2065(2)	40.7(16)
C48	1853(5)	-2876(5)	2680(3)	57(2)
C49	3328(4)	-1131(5)	1771(2)	45.0(18)
C50	2407(3)	-26(4)	2999.9(17)	21.9(12)

C51	3428(3)	-390(4)	2874.9(18)	24.9(12)
C52	3437(3)	-1070(4)	3175.5(19)	29.1(13)
C53	2883(3)	-1174(4)	3384.4(19)	25.4(12)
C54	4834(3)	-952(6)	3449(3)	47.3(19)
C55	4371(4)	-2330(5)	2785(3)	51(2)
C56	4060(4)	-2585(5)	3713(3)	53(2)
C57	672(3)	-820(3)	3889.0(16)	17.7(11)
C58	-350(3)	-1222(4)	3986.5(19)	27.4(13)
C59	-135(3)	-795(4)	4343.2(19)	26.5(13)
C60	30(2)	-1619(4)	3285.2(17)	22.3(12)
C61	-193(3)	-1065(4)	2943.1(18)	24.4(12)
C62	-353(3)	-1470(4)	2546.4(19)	29.4(13)
C63	-307(3)	-2383(4)	2490.4(19)	30.1(14)
C64	-73(3)	-2902(4)	2832(2)	29.7(13)
C65	97(3)	-2534(4)	3237(2)	28.2(13)
C66	-267(3)	-86(4)	3007(2)	29.5(13)
C67	-517(4)	-2803(5)	2059(2)	51(2)
C68	333(4)	-3118(4)	3612(2)	40.7(17)
C69	831(3)	72(4)	4567.4(17)	21.4(12)
C70	747(3)	987(4)	4491.0(17)	23.5(12)
C71	1074(3)	1582(4)	4770.3(18)	27.1(13)
C72	1477(3)	1291(4)	5110.5(19)	28.6(14)
C73	1532(3)	388(5)	5185.7(18)	28.5(14)
C74	1211(3)	-254(4)	4922.9(18)	24.9(12)
C75	325(3)	1319(4)	4114.2(19)	32.4(14)
C76	1852(3)	1964(5)	5396(2)	41.8(18)
C77	1260(3)	-1222(5)	5016(2)	37.5(16)
C78	2037(2)	1024(4)	4035.1(16)	18.7(11)
C79	2893(3)	1295(4)	4511.7(17)	22.3(12)
C80	2948(2)	399(4)	4601.3(17)	20.0(11)
C81	2516(3)	-159(4)	4383.7(17)	20.9(11)
C82	3988(3)	928(6)	5286(2)	45.6(18)
C83	4160(4)	-690(6)	4692(3)	55(2)
C84	3210(4)	-764(6)	5396(2)	49(2)
Cl1	3102.2(7)	3394.1(10)	4703.7(4)	29.8(3)
Cl2	4083.9(7)	1037.9(11)	2224.4(5)	30.6(3)
Cl3	1898.2(6)	-2098.6(9)	4022.3(4)	25.1(3)
Ir1	2302.3(2)	2998.2(2)	4087.3(2)	19.04(7)
Ir2	2972.1(2)	1397.5(2)	2418.7(2)	20.47(7)
Ir3	1501.5(2)	-730.0(2)	3629.0(2)	17.41(6)
N1	1636(2)	4757(3)	4163.6(15)	25.2(10)
N2	2394(2)	4894(3)	3741.2(15)	26.4(11)
N3	3069(2)	2733(3)	3648.9(14)	20.5(10)
N4	3328(2)	1951(3)	3022.1(14)	21.7(10)
N5	2573(2)	1565(3)	1479.0(14)	22.9(10)
N6	2377(2)	228(3)	1685.3(15)	27.9(11)
N7	2916(2)	134(3)	2782.6(14)	19.8(9)
N8	2360(2)	-657(3)	3298.5(14)	20.7(10)
N9	130(2)	-1239(3)	3708.9(15)	22(1)
N10	490(2)	-540(3)	4280.4(14)	21.3(10)
N11	2053.8(19)	141(3)	4090.8(14)	18.3(9)
N12	2423(2)	1627(3)	4218.5(15)	21(1)
Si1	5025.2(8)	2386.0(15)	3770.8(6)	37.9(4)

Si2	4186.1(8)	-1762.8(13)	3284.8(6)	34.3(4)
Si3	3597.3(8)	-48.5(13)	5002.1(5)	30.3(4)

Table S15. Anisotropic Displacement Parameters (Å^2×10^3) for $C_{84}H_{114}Cl_3Ir_3N_{12}Si_3$. The anisotropic displacement factor exponent takes the form: $-2\pi^2[h^2a^{*2}U_{11}+2hka^*b^*U_{12}+\ldots]$.

Atom	U_{11}	U_{22}	U_{33}	U_{23}	U_{13}	U_{12}
C1	26(3)	18(3)	12(2)	-5(2)	4(2)	-7(2)
C2	42(4)	24(3)	26(3)	4(2)	3(3)	6(3)
C3	42(4)	21(3)	27(3)	5(2)	-1(3)	2(3)
C4	39(3)	23(3)	26(3)	4(2)	13(3)	11(3)
C5	38(4)	23(3)	36(3)	3(3)	12(3)	7(3)
C6	39(4)	23(3)	61(5)	0(3)	15(3)	4(3)
C7	58(5)	24(4)	51(4)	9(3)	31(4)	16(3)
C8	80(6)	30(4)	27(3)	10(3)	29(4)	24(4)
C9	52(4)	31(4)	30(3)	-3(3)	11(3)	19(3)
C10	34(4)	42(4)	40(4)	-2(3)	-2(3)	-2(3)
C11	84(7)	44(5)	83(6)	14(5)	54(6)	17(5)
C12	67(5)	57(5)	25(3)	-3(3)	1(3)	21(4)
C13	33(3)	22(3)	30(3)	5(2)	7(3)	-2(3)
C14	50(4)	25(3)	31(3)	8(3)	10(3)	-4(3)
C15	59(5)	36(4)	34(4)	3(3)	15(3)	3(3)
C16	51(5)	63(6)	54(5)	15(4)	26(4)	13(4)
C17	32(4)	53(5)	56(5)	12(4)	3(3)	-3(3)
C18	36(4)	32(4)	41(4)	9(3)	9(3)	-8(3)
C19	47(4)	46(4)	28(3)	6(3)	-1(3)	-9(3)
C20	59(6)	108(9)	73(6)	8(6)	35(5)	13(6)
C21	42(4)	41(4)	48(4)	11(3)	-4(3)	-11(3)
C22	22(3)	27(3)	20(3)	5(2)	2(2)	4(2)
C23	26(3)	25(3)	25(3)	-3(2)	-4(2)	-2(2)
C24	23(3)	34(3)	27(3)	7(3)	-1(2)	-1(3)
C25	17(3)	36(3)	21(3)	4(2)	-1(2)	3(2)
C26	39(5)	163(12)	63(6)	40(7)	-2(4)	17(6)
C27	32(4)	74(6)	89(7)	-33(5)	3(4)	-11(4)
C28	23(3)	48(5)	71(5)	-2(4)	9(3)	4(3)
C29	22(3)	25(3)	20(3)	7(2)	-1(2)	5(2)
C30	49(4)	38(4)	24(3)	3(3)	-8(3)	1(3)
C31	51(4)	34(4)	22(3)	1(3)	-5(3)	0(3)
C32	23(3)	40(4)	12(2)	7(2)	-3(2)	3(3)
C33	28(3)	31(3)	22(3)	6(2)	-1(2)	3(3)
C34	39(4)	37(4)	26(3)	7(3)	1(3)	11(3)
C35	49(4)	35(4)	32(3)	2(3)	-2(3)	-7(3)
C36	33(4)	49(4)	31(3)	7(3)	-1(3)	-9(3)
C37	34(3)	36(4)	27(3)	8(3)	2(3)	-1(3)
C38	23(3)	41(4)	38(3)	14(3)	-4(3)	13(3)
C39	76(6)	42(5)	53(5)	1(4)	12(4)	-13(4)
C40	32(4)	50(4)	47(4)	14(4)	10(3)	-1(3)
C41	41(4)	24(3)	24(3)	1(2)	-3(3)	-2(3)
C42	43(4)	30(3)	23(3)	-2(3)	-1(3)	-2(3)
C43	46(4)	38(4)	30(3)	-4(3)	4(3)	-9(3)
C44	62(5)	33(4)	31(3)	4(3)	5(3)	-6(3)
C45	52(4)	28(4)	42(4)	0(3)	-3(3)	5(3)
C46	46(4)	30(4)	30(3)	-1(3)	2(3)	2(3)
C47	36(4)	41(4)	45(4)	-2(3)	1(3)	2(3)

C48	87(7)	32(4)	52(5)	11(4)	11(4)	-7(4)
C49	46(4)	44(4)	45(4)	3(3)	7(3)	13(3)
C50	18(3)	27(3)	20(3)	-1(2)	-1(2)	1(2)
C51	22(3)	28(3)	25(3)	4(2)	7(2)	7(2)
C52	26(3)	33(3)	29(3)	6(3)	7(2)	6(3)
C53	20(3)	27(3)	30(3)	4(2)	8(2)	5(2)
C54	25(3)	63(5)	54(4)	10(4)	1(3)	-1(3)
C55	49(5)	44(4)	62(5)	-2(4)	15(4)	20(4)
C56	55(5)	38(4)	67(5)	26(4)	7(4)	17(4)
C57	24(3)	11(3)	18(2)	3(2)	1(2)	1(2)
C58	16(3)	35(4)	32(3)	-7(3)	8(2)	-8(2)
C59	17(3)	39(4)	25(3)	-3(3)	13(2)	-4(2)
C60	13(2)	32(3)	21(3)	-6(2)	0(2)	-5(2)
C61	19(3)	31(3)	24(3)	1(2)	-1(2)	1(2)
C62	29(3)	35(4)	23(3)	1(3)	-6(2)	-1(3)
C63	27(3)	35(4)	27(3)	-8(3)	-4(2)	3(3)
C64	32(3)	21(3)	35(3)	-7(3)	-3(3)	3(3)
C65	21(3)	29(3)	33(3)	-1(3)	-5(2)	-1(2)
C66	28(3)	26(3)	33(3)	3(3)	-9(3)	-3(3)
C67	71(5)	47(5)	32(4)	-11(3)	-13(4)	4(4)
C68	47(4)	26(4)	46(4)	1(3)	-17(3)	-1(3)
C69	18(3)	28(3)	19(3)	-5(2)	3(2)	-5(2)
C70	27(3)	25(3)	19(3)	0(2)	4(2)	-2(2)
C71	32(3)	27(3)	23(3)	-3(2)	12(2)	-8(3)
C72	20(3)	43(4)	24(3)	-11(3)	7(2)	-11(3)
C73	15(3)	55(4)	15(3)	-4(3)	1(2)	1(3)
C74	16(3)	38(4)	21(3)	-5(2)	4(2)	1(2)
C75	39(4)	33(4)	25(3)	2(3)	-1(3)	6(3)
C76	34(4)	58(5)	34(4)	-17(3)	4(3)	-17(3)
C77	46(4)	39(4)	29(3)	2(3)	6(3)	9(3)
C78	15(2)	22(3)	18(2)	0(2)	-2(2)	3(2)
C79	15(3)	29(3)	22(3)	-1(2)	0(2)	-2(2)
C80	11(2)	28(3)	21(3)	2(2)	2(2)	3(2)
C81	20(3)	21(3)	23(3)	4(2)	5(2)	2(2)
C82	29(3)	71(5)	34(4)	0(4)	-17(3)	-8(4)
C83	47(4)	75(6)	44(4)	3(4)	2(4)	31(4)
C84	44(4)	61(5)	40(4)	19(4)	-5(3)	13(4)
Cl1	34.5(7)	29.3(8)	24.5(7)	-2.6(6)	-6.0(6)	-5.7(6)
Cl2	23.6(7)	39.7(8)	29.3(7)	3.6(6)	8.1(6)	3.7(6)
Cl3	25.2(7)	19.4(7)	31.2(7)	2.4(5)	6.0(5)	1.7(5)
Ir1	20.03(12)	17.28(12)	19.70(12)	-0.27(9)	0.78(9)	0.49(9)
Ir2	19.65(12)	24.90(13)	16.94(11)	3.36(9)	1.90(9)	4.16(9)
Ir3	14.21(11)	19.23(13)	18.97(11)	-0.47(9)	2.58(8)	-0.87(9)
N1	34(3)	20(3)	22(2)	0.0(19)	4(2)	5(2)
N2	28(3)	24(3)	28(2)	3(2)	5(2)	-1(2)
N3	21(2)	23(2)	18(2)	1.6(19)	1.7(18)	-2.0(19)
N4	20(2)	26(3)	19(2)	8.2(19)	-0.3(18)	4.8(19)
N5	23(2)	29(3)	15(2)	2.1(19)	-4.1(18)	6(2)
N6	36(3)	28(3)	20(2)	2(2)	-1(2)	0(2)
N7	19(2)	23(2)	18(2)	4.5(19)	4.6(18)	4.8(19)
N8	21(2)	20(2)	22(2)	2.0(19)	5.7(18)	3.2(19)
N9	19(2)	25(3)	22(2)	-6(2)	1.2(18)	-1.5(19)
N10	18(2)	25(3)	21(2)	-2.9(19)	3.1(18)	-5.8(19)

N11	9(2)	26(3)	20(2)	-3.6(19)	-4.9(16)	-0.9(18)
N12	22(2)	16(2)	26(2)	-8.2(19)	7.5(19)	-2.5(19)
Si1	19.4(8)	61.8(13)	32.1(9)	5.6(9)	-0.2(7)	1.8(8)
Si2	25.8(8)	36.1(10)	42.1(10)	12.6(8)	9.9(7)	11.5(7)
Si3	19.3(8)	45.1(11)	25.7(8)	5.0(7)	-3.4(6)	6.5(7)

Table S16. Bond Lengths for $C_{84}H_{114}Cl_3Ir_3N_{12}Si_3$.

Atom	Atom	Length/Å	Atom	Atom	Length/Å
C1	Ir1	1.973(5)	C44	C48	1.526(9)
C1	N1	1.385(7)	C45	C46	1.399(10)
C1	N2	1.361(7)	C46	C49	1.489(10)
C2	C3	1.333(9)	C50	N7	1.327(7)
C2	N1	1.389(8)	C50	N8	1.333(7)
C3	N2	1.379(8)	C51	C52	1.382(8)
C4	C5	1.390(10)	C51	N7	1.345(7)
C4	C9	1.397(9)	C52	C53	1.382(8)
C4	N1	1.431(8)	C52	Si2	1.894(6)
C5	C6	1.404(9)	C53	N8	1.354(7)
C5	C10	1.509(9)	C54	Si2	1.867(8)
C6	C7	1.380(11)	C55	Si2	1.839(8)
C7	C8	1.383(12)	C56	Si2	1.847(7)
C7	C11	1.491(10)	C57	Ir3	1.978(5)
C8	C9	1.398(10)	C57	N9	1.382(7)
C9	C12	1.489(11)	C57	N10	1.368(7)
C13	C14	1.398(9)	C58	C59	1.331(8)
C13	C18	1.392(9)	C58	N9	1.374(7)
C13	N2	1.437(7)	C59	N10	1.393(7)
C14	C15	1.379(9)	C60	C61	1.403(8)
C14	C19	1.497(10)	C60	C65	1.387(9)
C15	C16	1.396(12)	C60	N9	1.436(7)
C16	C17	1.375(12)	C61	C62	1.394(8)
C16	C20	1.509(10)	C61	C66	1.488(8)
C17	C18	1.395(10)	C62	C63	1.383(9)
C18	C21	1.498(10)	C63	C64	1.378(9)
C22	N3	1.343(7)	C63	C67	1.517(8)
C22	N4	1.339(7)	C64	C65	1.399(8)
C23	C24	1.392(8)	C65	C68	1.512(9)
C23	N3	1.345(7)	C69	C70	1.399(8)
C24	C25	1.418(8)	C69	C74	1.403(8)
C24	Si1	1.877(6)	C69	N10	1.435(7)
C25	N4	1.335(7)	C70	C71	1.390(8)
C26	Si1	1.854(9)	C70	C75	1.502(8)
C27	Si1	1.862(9)	C71	C72	1.377(9)
C28	Si1	1.853(7)	C72	C73	1.377(9)
C29	Ir2	1.978(6)	C72	C76	1.524(8)
C29	N5	1.374(7)	C73	C74	1.402(8)
C29	N6	1.377(8)	C74	C77	1.480(9)
C30	C31	1.337(10)	C78	N11	1.334(7)
C30	N5	1.394(8)	C78	N12	1.316(7)
C31	N6	1.392(7)	C79	C80	1.375(8)
C32	C33	1.390(8)	C79	N12	1.386(7)
C32	C37	1.385(8)	C80	C81	1.374(8)

C32	N5	1.433(8)	C80	Si3	1.901(5)
C33	C34	1.381(9)	C81	N11	1.358(7)
C33	C38	1.512(9)	C82	Si3	1.868(7)
C34	C35	1.394(10)	C83	Si3	1.848(7)
C35	C36	1.382(10)	C84	Si3	1.858(7)
C35	C39	1.527(10)	Cl1	Ir1	2.5264(14)
C36	C37	1.396(10)	Cl2	Ir2	2.5118(14)
C37	C40	1.507(9)	Cl3	Ir3	2.4990(13)
C41	C42	1.399(9)	Ir1	N3	2.219(4)
C41	C46	1.404(9)	Ir1	N12	2.106(5)
C41	N6	1.435(8)	Ir2	N4	2.136(5)
C42	C43	1.394(9)	Ir2	N7	2.213(4)
C42	C47	1.493(9)	Ir3	N8	2.142(4)
C43	C44	1.394(10)	Ir3	N11	2.207(4)
C44	C45	1.381(10)			

Table S17. Bond Angles for $C_{84}H_{114}Cl_3Ir_3N_{12}Si_3$.

Atom	Atom	Atom	Angle/˚	Atom	Atom	Atom	Angle/˚
N1	C1	Ir1	125.8(4)	C60	C65	C68	121.3(5)
N2	C1	Ir1	131.3(4)	C64	C65	C68	121.0(6)
N2	C1	N1	102.9(5)	C70	C69	C74	122.1(5)
C3	C2	N1	107.1(5)	C70	C69	N10	117.9(5)
C2	C3	N2	107.1(5)	C74	C69	N10	119.9(5)
C5	C4	C9	121.1(6)	C69	C70	C75	121.1(5)
C5	C4	N1	119.4(5)	C71	C70	C69	118.1(5)
C9	C4	N1	119.3(6)	C71	C70	C75	120.8(6)
C4	C5	C6	119.7(6)	C72	C71	C70	121.7(6)
C4	C5	C10	120.2(6)	C71	C72	C73	118.7(5)
C6	C5	C10	120.1(6)	C71	C72	C76	120.1(6)
C7	C6	C5	120.6(7)	C73	C72	C76	121.1(6)
C6	C7	C8	118.1(6)	C72	C73	C74	122.9(6)
C6	C7	C11	122.3(8)	C69	C74	C77	121.5(5)
C8	C7	C11	119.6(8)	C73	C74	C69	116.3(6)
C7	C8	C9	123.6(6)	C73	C74	C77	122.3(6)
C4	C9	C8	116.8(7)	N12	C78	N11	127.9(5)
C4	C9	C12	119.5(6)	C80	C79	N12	121.9(5)
C8	C9	C12	123.7(6)	C79	C80	Si3	121.5(4)
C14	C13	N2	118.6(6)	C81	C80	C79	116.8(5)
C18	C13	C14	122.5(6)	C81	C80	Si3	121.7(4)
C18	C13	N2	118.9(6)	N11	C81	C80	122.9(5)
C13	C14	C19	121.5(6)	C1	Ir1	Cl1	90.56(15)
C15	C14	C13	117.7(7)	C1	Ir1	N3	102.77(19)
C15	C14	C19	120.8(6)	C1	Ir1	N12	174.85(19)
C14	C15	C16	121.9(7)	N3	Ir1	Cl1	92.09(12)
C15	C16	C20	120.0(8)	N12	Ir1	Cl1	91.02(13)
C17	C16	C15	118.3(7)	N12	Ir1	N3	82.07(17)
C17	C16	C20	121.6(8)	C29	Ir2	Cl2	90.92(16)
C16	C17	C18	122.5(7)	C29	Ir2	N4	173.2(2)
C13	C18	C17	117.1(6)	C29	Ir2	N7	101.4(2)
C13	C18	C21	121.6(6)	N4	Ir2	Cl2	91.31(13)
C17	C18	C21	121.4(7)	N4	Ir2	N7	84.88(17)
N4	C22	N3	124.3(5)	N7	Ir2	Cl2	91.39(12)

N3	C23	C24	123.7(5)	C57	Ir3	Cl3	90.92(15)
C23	C24	C25	114.5(5)	C57	Ir3	N8	175.37(19)
C23	C24	Si1	120.8(5)	C57	Ir3	N11	102.21(19)
C25	C24	Si1	124.6(5)	N8	Ir3	Cl3	90.84(12)
N4	C25	C24	122.1(5)	N8	Ir3	N11	82.03(17)
N5	C29	Ir2	125.5(4)	N11	Ir3	Cl3	91.47(12)
N5	C29	N6	102.9(5)	C1	N1	C2	110.8(5)
N6	C29	Ir2	131.6(4)	C1	N1	C4	127.6(5)
C31	C30	N5	107.9(5)	C2	N1	C4	121.5(5)
C30	C31	N6	106.1(6)	C1	N2	C3	112.1(5)
C33	C32	N5	117.2(5)	C1	N2	C13	126.5(5)
C37	C32	C33	121.8(6)	C3	N2	C13	121.2(5)
C37	C32	N5	121.0(5)	C22	N3	C23	117.0(5)
C32	C33	C38	120.6(6)	C22	N3	Ir1	118.2(4)
C34	C33	C32	119.0(6)	C23	N3	Ir1	122.9(4)
C34	C33	C38	120.5(6)	C22	N4	Ir2	118.0(4)
C33	C34	C35	120.5(6)	C25	N4	C22	118.5(5)
C34	C35	C39	119.6(7)	C25	N4	Ir2	123.5(4)
C36	C35	C34	119.3(6)	C29	N5	C30	111.0(5)
C36	C35	C39	121.1(7)	C29	N5	C32	125.9(5)
C35	C36	C37	121.3(6)	C30	N5	C32	122.2(5)
C32	C37	C36	118.0(6)	C29	N6	C31	112.0(5)
C32	C37	C40	120.8(6)	C29	N6	C41	126.6(5)
C36	C37	C40	121.2(6)	C31	N6	C41	121.1(5)
C42	C41	C46	122.4(6)	C50	N7	C51	116.5(5)
C42	C41	N6	119.5(6)	C50	N7	Ir2	119.3(4)
C46	C41	N6	118.0(6)	C51	N7	Ir2	122.5(4)
C41	C42	C47	121.9(6)	C50	N8	C53	116.3(5)
C43	C42	C41	118.1(6)	C50	N8	Ir3	118.6(4)
C43	C42	C47	120.0(6)	C53	N8	Ir3	125.1(4)
C42	C43	C44	121.3(7)	C57	N9	C60	127.8(4)
C43	C44	C48	119.2(7)	C58	N9	C57	110.9(4)
C45	C44	C43	118.9(6)	C58	N9	C60	121.3(5)
C45	C44	C48	121.9(7)	C57	N10	C59	111.5(5)
C44	C45	C46	122.6(7)	C57	N10	C69	126.2(4)
C41	C46	C49	121.2(6)	C59	N10	C69	121.4(5)
C45	C46	C41	116.7(6)	C78	N11	C81	115.2(5)
C45	C46	C49	122.1(6)	C78	N11	Ir3	119.6(3)
N7	C50	N8	125.9(5)	C81	N11	Ir3	123.8(4)
N7	C51	C52	123.1(5)	C78	N12	C79	115.2(5)
C51	C52	C53	115.5(5)	C78	N12	Ir1	121.6(4)
C51	C52	Si2	119.7(4)	C79	N12	Ir1	123.2(4)
C53	C52	Si2	124.8(5)	C26	Si1	C24	106.8(3)
N8	C53	C52	122.8(5)	C26	Si1	C27	108.8(5)
N9	C57	Ir3	126.1(4)	C27	Si1	C24	108.3(3)
N10	C57	Ir3	130.8(4)	C28	Si1	C24	109.1(3)
N10	C57	N9	103.0(4)	C28	Si1	C26	112.4(5)
C59	C58	N9	108.2(5)	C28	Si1	C27	111.3(4)
C58	C59	N10	106.3(5)	C54	Si2	C52	105.8(3)
C61	C60	N9	118.7(5)	C55	Si2	C52	108.9(3)
C65	C60	C61	122.3(5)	C55	Si2	C54	109.7(4)
C65	C60	N9	118.7(5)	C55	Si2	C56	110.6(4)
C60	C61	C66	120.9(5)	C56	Si2	C52	109.6(3)

C62	C61	C60	117.3(6)	C56	Si2	C54	112.1(4)
C62	C61	C66	121.7(5)	C82	Si3	C80	107.7(3)
C63	C62	C61	121.9(6)	C83	Si3	C80	107.2(3)
C62	C63	C67	120.2(6)	C83	Si3	C82	112.2(4)
C64	C63	C62	119.0(5)	C83	Si3	C84	111.8(4)
C64	C63	C67	120.8(6)	C84	Si3	C80	107.9(3)
C63	C64	C65	121.7(6)	C84	Si3	C82	109.8(4)
C60	C65	C64	117.7(6)				

Table S18. Torsion Angles for $C_{84}H_{114}Cl_3Ir_3N_{12}Si_3$.

A	B	C	D	Angle/˚	A	B	C	D	Angle/˚
C2	C3	N2	C1	-2.2(7)	C61	C62	C63	C67	177.0(6)
C2	C3	N2	C13	173.9(5)	C62	C63	C64	C65	2.5(10)
C3	C2	N1	C1	1.8(7)	C63	C64	C65	C60	-0.9(9)
C3	C2	N1	C4	-174.4(5)	C63	C64	C65	C68	177.9(6)
C4	C5	C6	C7	1.1(10)	C65	C60	C61	C62	0.5(8)
C5	C4	C9	C8	2.1(9)	C65	C60	C61	C66	179.2(5)
C5	C4	C9	C12	-176.7(6)	C65	C60	N9	C57	99.2(7)
C5	C4	N1	C1	-86.9(7)	C65	C60	N9	C58	-84.0(7)
C5	C4	N1	C2	88.7(7)	C66	C61	C62	C63	-177.5(6)
C5	C6	C7	C8	1.0(10)	C67	C63	C64	C65	-177.2(6)
C5	C6	C7	C11	-179.5(6)	C69	C70	C71	C72	0.8(8)
C6	C7	C8	C9	-1.7(10)	C70	C69	C74	C73	-3.5(8)
C7	C8	C9	C4	0.2(10)	C70	C69	C74	C77	175.8(5)
C7	C8	C9	C12	178.9(7)	C70	C69	N10	C57	83.0(7)
C9	C4	C5	C6	-2.8(9)	C70	C69	N10	C59	-85.4(7)
C9	C4	C5	C10	178.7(6)	C70	C71	C72	C73	-3.1(8)
C9	C4	N1	C1	97.0(7)	C70	C71	C72	C76	176.9(5)
C9	C4	N1	C2	-87.4(7)	C71	C72	C73	C74	2.1(8)
C10	C5	C6	C7	179.6(6)	C72	C73	C74	C69	1.1(8)
C11	C7	C8	C9	178.8(6)	C72	C73	C74	C77	-178.1(5)
C13	C14	C15	C16	-2.7(10)	C74	C69	C70	C71	2.6(8)
C14	C13	C18	C17	0.7(10)	C74	C69	C70	C75	-178.4(5)
C14	C13	C18	C21	-177.7(6)	C74	C69	N10	C57	-99.9(6)
C14	C13	N2	C1	82.1(8)	C74	C69	N10	C59	91.6(7)
C14	C13	N2	C3	-93.4(7)	C75	C70	C71	C72	-178.2(5)
C14	C15	C16	C17	2.1(12)	C76	C72	C73	C74	-177.9(5)
C14	C15	C16	C20	-179.9(8)	C79	C80	C81	N11	0.4(8)
C15	C16	C17	C18	0.1(12)	C80	C79	N12	C78	0.2(7)
C16	C17	C18	C13	-1.4(11)	C80	C79	N12	Ir1	177.9(4)
C16	C17	C18	C21	177.0(7)	C80	C81	N11	C78	-1.6(7)
C18	C13	C14	C15	1.3(10)	C80	C81	N11	Ir3	165.2(4)
C18	C13	C14	C19	-179.1(6)	Ir1	C1	N1	C2	176.5(4)
C18	C13	N2	C1	-101.2(7)	Ir1	C1	N1	C4	-7.5(8)
C18	C13	N2	C3	83.4(7)	Ir1	C1	N2	C3	-176.3(4)
C19	C14	C15	C16	177.7(7)	Ir1	C1	N2	C13	7.9(9)
C20	C16	C17	C18	-177.9(8)	Ir2	C29	N5	C30	177.0(4)
C23	C24	C25	N4	0.9(9)	Ir2	C29	N5	C32	-13.6(8)
C23	C24	Si1	C26	54.1(7)	Ir2	C29	N6	C31	-177.1(5)
C23	C24	Si1	C27	-62.9(6)	Ir2	C29	N6	C41	8.8(9)
C23	C24	Si1	C28	175.9(5)	Ir3	C57	N9	C58	176.5(4)
C24	C23	N3	C22	-1.3(9)	Ir3	C57	N9	C60	-6.5(8)

C24	C23	N3	Ir1	162.6(5)	Ir3	C57	N10	C59	-176.0(4)
C24	C25	N4	C22	-1.2(9)	Ir3	C57	N10	C69	14.6(8)
C24	C25	N4	Ir2	176.8(4)	N1	C1	N2	C3	3.1(6)
C25	C24	Si1	C26	-123.6(7)	N1	C1	N2	C13	-172.7(5)
C25	C24	Si1	C27	119.4(6)	N1	C2	C3	N2	0.2(7)
C25	C24	Si1	C28	-1.9(6)	N1	C4	C5	C6	-178.8(5)
C30	C31	N6	C29	-0.7(8)	N1	C4	C5	C10	2.7(9)
C30	C31	N6	C41	173.7(6)	N1	C4	C9	C8	178.1(5)
C31	C30	N5	C29	1.3(8)	N1	C4	C9	C12	-0.7(9)
C31	C30	N5	C32	-168.5(6)	N2	C1	N1	C2	-3.0(6)
C32	C33	C34	C35	-1.7(9)	N2	C1	N1	C4	173.0(5)
C33	C32	C37	C36	2.7(9)	N2	C13	C14	C15	178.0(6)
C33	C32	C37	C40	-179.5(6)	N2	C13	C14	C19	-2.4(9)
C33	C32	N5	C29	-75.5(7)	N2	C13	C18	C17	-176.0(6)
C33	C32	N5	C30	92.8(7)	N2	C13	C18	C21	5.7(9)
C33	C34	C35	C36	3.9(10)	N3	C22	N4	C25	0.3(8)
C33	C34	C35	C39	-175.8(6)	N3	C22	N4	Ir2	-177.8(4)
C34	C35	C36	C37	-2.8(10)	N3	C23	C24	C25	0.5(9)
C35	C36	C37	C32	-0.4(10)	N3	C23	C24	Si1	-177.5(5)
C35	C36	C37	C40	-178.2(6)	N4	C22	N3	C23	1.0(8)
C37	C32	C33	C34	-1.7(9)	N4	C22	N3	Ir1	-163.7(4)
C37	C32	C33	C38	179.7(5)	N5	C29	N6	C31	1.5(7)
C37	C32	N5	C29	104.7(7)	N5	C29	N6	C41	-172.6(5)
C37	C32	N5	C30	-87.0(7)	N5	C30	C31	N6	-0.3(8)
C38	C33	C34	C35	176.9(6)	N5	C32	C33	C34	178.6(5)
C39	C35	C36	C37	176.8(7)	N5	C32	C33	C38	-0.1(8)
C41	C42	C43	C44	0.1(10)	N5	C32	C37	C36	-177.5(5)
C42	C41	C46	C45	-1.8(10)	N5	C32	C37	C40	0.3(9)
C42	C41	C46	C49	177.4(6)	N6	C29	N5	C30	-1.7(6)
C42	C41	N6	C29	86.9(8)	N6	C29	N5	C32	167.7(5)
C42	C41	N6	C31	-86.7(8)	N6	C41	C42	C43	178.4(5)
C42	C43	C44	C45	-2.2(10)	N6	C41	C42	C47	-3.6(9)
C42	C43	C44	C48	176.8(7)	N6	C41	C46	C45	-178.3(6)
C43	C44	C45	C46	2.4(11)	N6	C41	C46	C49	0.9(9)
C44	C45	C46	C41	-0.4(10)	N7	C50	N8	C53	0.1(8)
C44	C45	C46	C49	-179.6(7)	N7	C50	N8	Ir3	176.5(4)
C46	C41	C42	C43	2.0(9)	N7	C51	C52	C53	-1.0(9)
C46	C41	C42	C47	-180.0(6)	N7	C51	C52	Si2	-179.5(5)
C46	C41	N6	C29	-96.5(7)	N8	C50	N7	C51	-0.1(8)
C46	C41	N6	C31	89.9(8)	N8	C50	N7	Ir2	-165.7(4)
C47	C42	C43	C44	-178.0(6)	N9	C57	N10	C59	1.4(6)
C48	C44	C45	C46	-176.6(7)	N9	C57	N10	C69	-168.0(5)
C51	C52	C53	N8	1.0(9)	N9	C58	C59	N10	0.4(7)
C51	C52	Si2	C54	56.9(6)	N9	C60	C61	C62	-173.0(5)
C51	C52	Si2	C55	-60.9(6)	N9	C60	C61	C66	5.7(8)
C51	C52	Si2	C56	178.0(6)	N9	C60	C65	C64	172.9(5)
C52	C51	N7	C50	0.6(9)	N9	C60	C65	C68	-5.9(8)
C52	C51	N7	Ir2	165.7(5)	N10	C57	N9	C58	-1.1(6)
C52	C53	N8	C50	-0.5(9)	N10	C57	N9	C60	175.9(5)
C52	C53	N8	Ir3	-176.7(5)	N10	C69	C70	C71	179.6(5)
C53	C52	Si2	C54	-121.4(6)	N10	C69	C70	C75	-1.5(8)
C53	C52	Si2	C55	120.8(6)	N10	C69	C74	C73	179.6(5)
C53	C52	Si2	C56	-0.4(7)	N10	C69	C74	C77	-1.2(8)

C58	C59	N10	C57	-1.2(7)	N11	C78	N12	C79	-1.7(8)
C58	C59	N10	C69	168.8(5)	N11	C78	N12	Ir1	-179.4(4)
C59	C58	N9	C57	0.5(7)	N12	C78	N11	C81	2.3(8)
C59	C58	N9	C60	-176.8(5)	N12	C78	N11	Ir3	-165.0(4)
C60	C61	C62	C63	1.2(9)	N12	C79	C80	C81	0.4(8)
C61	C60	C65	C64	-0.6(9)	N12	C79	C80	Si3	179.7(4)
C61	C60	C65	C68	-179.4(6)	Si1	C24	C25	N4	178.7(4)
C61	C60	N9	C57	-87.0(7)	Si2	C52	C53	N8	179.4(5)
C61	C60	N9	C58	89.8(7)	Si3	C80	C81	N11	-178.9(4)
C61	C62	C63	C64	-2.7(9)					

Table S19. Hydrogen Atom Coordinates (Å×10[4]) and Isotropic Displacement Parameters (Å[2]×10[3]) for $C_{84}H_{114}Cl_3Ir_3N_{12}Si_3$.

Atom	*x*	*y*	*z*	U(eq)
H2	1395	6105	4129	37
H3	2259	6253	3652	36
H6	-290	3591	4315	49
H8	765	4177	5392	53
H10A	538	4691	3612	58
H10B	76	3872	3633	58
H10C	811	3717	3613	58
H11A	-261	2875	5249	101
H11B	-756	3522	5011	101
H11C	-352	3843	5426	101
H12A	2171	4435	5041	75
H12B	1776	4940	5374	75
H12C	1919	5402	4939	75
H15	3043	4076	2475	51
H17	4384	4949	3347	56
H19A	1842	3796	3086	60
H19B	1968	4017	2607	60
H19C	1757	4775	2914	60
H20A	4643	4760	2602	118
H20B	4106	4470	2254	118
H20C	4442	3754	2560	118
H21A	3473	5745	4093	66
H21B	4155	5343	4052	66
H21C	3614	4750	4225	66
H22	2480	2326	3172	28
H23	3814	3035	4040	31
H25	4237	1682	2991	29
H26A	4726	2161	4478	133
H26B	5461	1992	4464	133
H26C	4972	1259	4295	133
H27A	5299	3826	3536	97
H27B	5709	3597	3963	97
H27C	4996	3901	3980	97
H28A	5367	1144	3385	71
H28B	5953	1762	3512	71
H28C	5473	2017	3122	71
H30	2267	1312	839	45
H31	2037	-205	1073	43
H34	1873	4297	1696	41

H36	3623	4102	1273	45
H38A	1381	2189	1527	52
H38B	1239	3036	1800	52
H38C	1652	2256	2009	52
H39A	2449	5647	1458	85
H39B	3171	5563	1367	85
H39C	2968	5513	1841	85
H40A	3919	1987	1434	64
H40B	4060	2705	1086	64
H40C	3573	1934	970	64
H43	1143	-1464	2462	46
H45	2862	-2436	2244	49
H47A	1334	634	2194	61
H47B	792	-83	2208	61
H47C	1045	196	1765	61
H48A	1810	-2686	2971	85
H48B	2198	-3296	2675	85
H48C	1464	-3155	2569	85
H49A	3246	-1095	1464	67
H49B	3591	-1641	1843	67
H49C	3543	-599	1875	67
H50	2051	333	2938	26
H51	3793	-290	2730	30
H53	2868	-1617	3593	30
H54A	4839	-483	3239	71
H54B	5239	-1255	3467	71
H54C	4759	-702	3725	71
H55A	4038	-2747	2702	77
H55B	4769	-2643	2831	77
H55C	4403	-1896	2560	77
H56A	3917	-2283	3960	79
H56B	4455	-2888	3792	79
H56C	3745	-3012	3609	79
H58	-755	-1467	3934	33
H59	-357	-688	4586	32
H62	-494	-1116	2313	35
H64	-26	-3513	2792	36
H66A	-528	18	3243	44
H66B	-466	175	2749	44
H66C	145	179	3070	44
H67A	-868	-2472	1925	77
H67B	-646	-3409	2103	77
H67C	-167	-2795	1877	77
H68A	706	-2854	3756	61
H68B	437	-3699	3507	61
H68C	5	-3172	3810	61
H71	1019	2192	4726	32
H73	1794	194	5421	34
H75A	-104	1116	4140	49
H75B	477	1094	3852	49
H75C	332	1960	4110	49
H76A	2137	2291	5226	63
H76B	2095	1654	5623	63

H76C	1562	2370	5519	63
H77A	860	-1434	5108	56
H77B	1591	-1324	5239	56
H77C	1359	-1535	4760	56
H78	1712	1240	3843	22
H79	3179	1691	4652	27
H81	2541	-768	4439	25
H82A	4195	1289	5083	68
H82B	4298	720	5505	68
H82C	3671	1278	5417	68
H83A	3942	-1190	4556	83
H83B	4508	-899	4883	83
H83C	4322	-313	4475	83
H84A	2867	-440	5512	73
H84B	3518	-928	5625	73
H84C	3044	-1292	5253	73
H	1706	3020	4405	29
HA	1813	2668	3679	29
HB	2237	1614	2544	31
HC	3031	2415	2222	31
HD	1123	-1290	3232	26
HE	1284	213	3387	26

Table S20. Solvent masks information for s $C_{84}H_{114}Cl_3Ir_3N_{12}Si_3$.

Number	x	y	z	Volume	Electron count	Content
1	0.500	0.000	0.000	764.6	98.6	?
2	-0.500	0.500	0.500	764.6	98.6	?
3	0.377	0.047	0.380	15.9	0.0	?
4	0.377	0.453	0.880	15.9	0.0	?
5	0.623	0.547	0.120	15.9	0.0	?
6	0.623	0.953	0.620	15.9	0.0	?

2.8 Complex characterisation by XRD – 3b

2.8.1 Experimental: Single crystals of $C_{89.42}H_{141.68}Cl_3Ir_3N_{12}O_{5.42}Si_3$ were prepared in methanol-d_4, under 3 bar H_2. A suitable crystal was selected, immersed in oil and placed on the micromount of a SuperNova, Dual, Cu at zero, Eos diffractometer. The crystal was kept at 110.05(10) K during data collection. Using Olex2 [16], the structure was solved with the ShelXS [17] structure solution program using Direct Methods and refined with the ShelXL [18] refinement package using Least Squares minimisation.

2.8.2 Crystal structure determination of $C_{89.42}H_{141.68}Cl_3Ir_3N_{12}O_{5.42}Si_3$.

Crystal data for $C_{89.41875}H_{141.67625}Cl_3Ir_3N_{12}O_{5.41875}Si_3$ (M =2238.76 g/mol): cubic, space group Pa-3 (no. 205), a = 27.98535(15) Å, V = 21917.6(3) Å^3, Z = 8, T = 110.05(10) K, μ(MoKα) = 3.788 mm^{-1}, $Dcalc$ = 1.357 g/cm^3, 61051 reflections measured (6.672° ≤ 2Θ ≤ 64.236°), 12096 unique (R_{int} = 0.0379, R_{sigma} = 0.0314) which were used in all calculations. The final R_1 was 0.0439 (I > 2σ(I)) and wR_2 was 0.1136 (all data).

2.8.3 Refinement model description:

Number of restraints - 6, number of constraints - unknown.

Details:

1. Fixed Uiso

At 1.2 times of: all C(H) groups, {H17A,H17B,H17C} of C17, All C(H,H) groups.
At 1.5 times of: all O(H) groups, {H30A,H30B,H30C} of C30, {H29A,H29B,H29C} of C29, {H28A, H28B,H28C} of C28, {H27A,H27B,H27C} of C27, {H26A,H26B,H26C} of C26, {H31A, H31B,H31C} of C31, {H5A,H5B,H5C} of C5, {H6A,H6B,H6C} of C6, {H7A,H7B,H7C} of C7, {H18A,H18B,H18C} of C18, {H19A,H19B,H19C} of C19

2. Uiso/Uaniso restraints and constraints: Uanis(N2) ≈ Ueq: with sigma of 0.001 and sigma for terminal atoms of 0.002

3. Others:
Sof(C31)=Sof(H31A)=Sof(H31B)=Sof(H31C)=Sof(O3)=Sof(H3A)=FVAR(1).
Sof(C30)=Sof(H30A)=Sof(H30B)=Sof(H30C)=Sof(O2)=Sof(H2)=FVAR(2).
Fixed Sof: H29A(0.33333) H29B(0.33333) H29C(0.33333) H1A(0.33333).

4.a Secondary CH2 refined with riding coordinates: C9(H9A,H9B), C10(H10A,H10B)
4.b Aromatic/amide H refined with riding coordinates: C1(H1), C3(H3), C4(H4), C13(H13), C15(H15), C22(H22), C24(H24)
4.c Idealised Me refined as rotating group:
C5(H5A,H5B,H5C), C6(H6A,H6B,H6C), C7(H7A,H7B,H7C), C17(H17A,H17B,H17C), C18(H18A,H18B,H18C), C19(H19A,H19B,H19C), C26(H26A,H26B,H26C), C27(H27A,H27B, H27C), C28(H28A,H28B,H28C), C29(H29A,H29B,H29C), C30(H30A,H30B,H30C), C31(H31A, H31B,H31C)
4.d Idealised tetrahedral OH refined with riding coordinates: O3(H3A).
4.e Idealised tetrahedral OH refined as rotating group: O1(H1A), O2(H2).

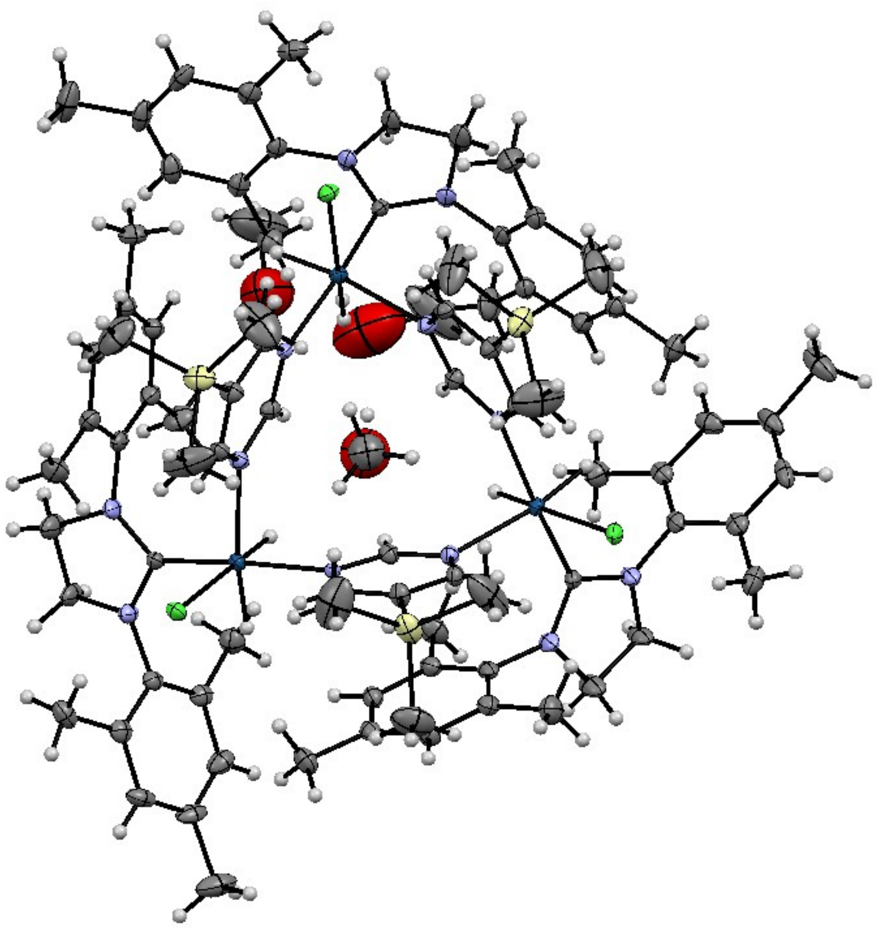

Figure S14. XRD structure of $C_{89.41875}H_{141.67625}Cl_3Ir_3N_{12}O_5$.

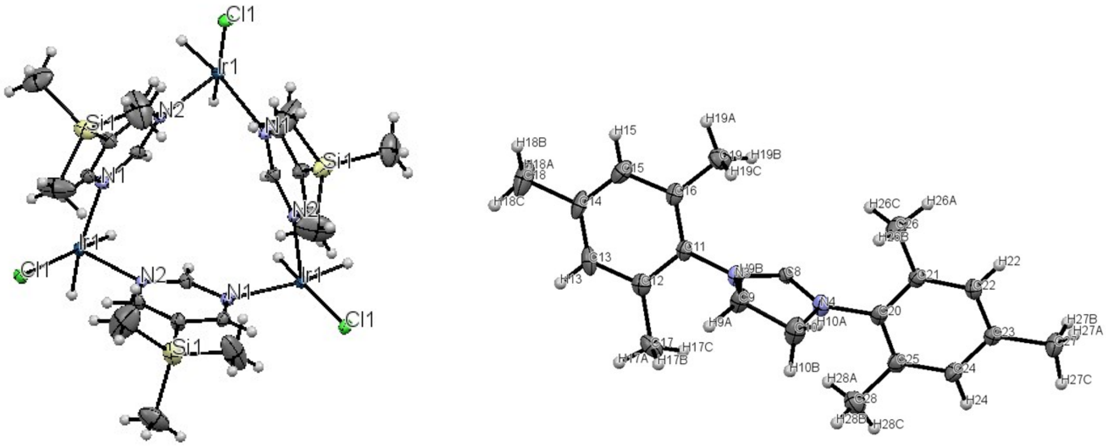

a). **b).**

Figure S15. Selected aspects of the XRD structure of $C_{89.41875}H_{141.67625}Cl_3Ir_3N_{12}O_5$**: a). Representation of central trimer without the three carbene units attached to the Ir atoms; b). Representation of the carbine unit with the corresponding atom labels.**

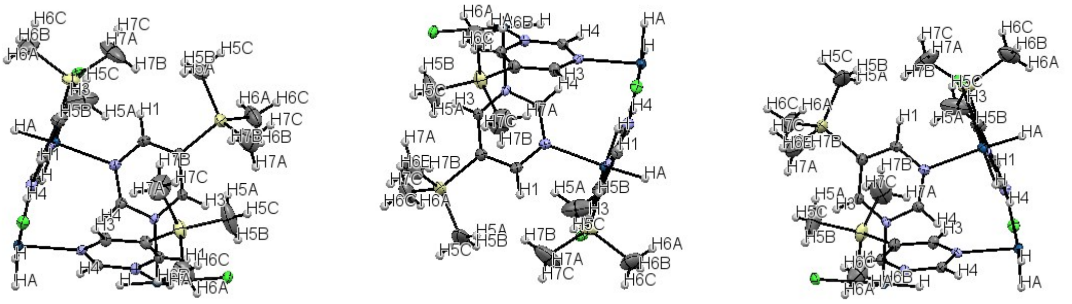

Figure S16. Selected aspects of the XRD structure of $C_{89.41875}H_{141.67625}Cl_3Ir_3N_{12}O_{5.:}$ Representation of central trimer without the three carbene units attached to the Ir atoms and the corresponding labels for the H atoms. For clarity the trimer is presented from three different perspectives.

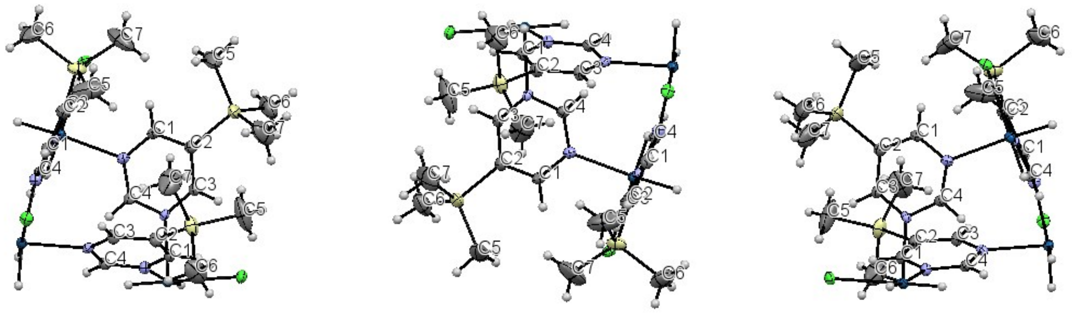

Figure S17. Selected aspects of the XRD structure of $C_{89.41875}H_{141.67625}Cl_3Ir_3N_{12}O_{5.}$: Representation of central trimer without the three carbene units attached to the Ir atoms and the corresponding labels for the C atoms. For clarity the trimer is presented from three different perspectives.

Table S21. Fractional Atomic Coordinates (×10[4]) and Equivalent Isotropic Displacement Parameters (Å[2]×10[3]) for $C_{89.41875}H_{141.67625}Cl_3Ir_3N_{12}O_5$.. U_{eq} is defined as 1/3 of of the trace of the orthogonalised U_{IJ} tensor.

Atom	*x*	*y*	*z*	U(eq)
C1	3173.8(18)	6639.2(18)	3063.6(17)	18.0(9)
C2	3605.4(18)	6803.1(18)	2878.6(19)	20.6(10)
C3	3644.2(17)	7298.0(17)	2843.5(18)	18.1(9)
C4	2894.4(17)	7400.1(17)	3158.1(17)	16.5(8)
C5	3859(3)	5787(3)	2582(4)	68(3)
C6	4513(3)	6342(3)	3247(3)	54(2)
C7	4444(3)	6635(4)	2207(4)	71(3)
C8	2066.2(17)	6633.3(16)	4065.0(16)	15.1(8)
C9	1769(2)	6534(2)	4857.3(18)	26.1(12)
C10	2293(2)	6672(3)	4882.4(19)	33.1(14)
C11	1262.4(18)	6242.4(19)	4197.0(17)	18.7(9)
C12	1214(2)	5746(2)	4220.0(18)	22.5(10)
C13	770(2)	5543(2)	4115.6(19)	27.3(12)
C14	383(2)	5821(2)	3989(2)	28.0(12)
C15	434(2)	6319(2)	3990(2)	27.8(12)
C16	870.0(19)	6535.7(19)	4098.3(18)	20.8(10)
C17	1633(2)	5438(2)	4366(2)	28.3(12)
C18	-83(2)	5592(3)	3838(3)	44.7(18)
C19	919(2)	7071(2)	4106(2)	28.3(11)
C20	2834.5(18)	7033(2)	4284.1(17)	20.1(10)
C21	2827.9(18)	7533(2)	4293.0(18)	21.1(10)
C22	3254.5(18)	7774.6(19)	4228.8(18)	20.8(10)
C23	3683.4(18)	7529.5(19)	4155.3(18)	19.6(9)
C24	3677.4(19)	7031(2)	4160(2)	23.2(10)
C25	3257.9(19)	6776(2)	4226.3(19)	22.4(10)
C26	2365(2)	7802(2)	4379(3)	34.3(14)
C27	4139(2)	7800(2)	4057(2)	28.3(12)
C28	3257(2)	6237(2)	4232(2)	34.6(14)
C29	1608(3)	8392(3)	3392(3)	54(3)
C30	1321(7)	8443(7)	4933(5)	109(8)
Cl1	2367.3(4)	5804.8(4)	3306.1(4)	18.9(2)
Ir1	2082.8(2)	6659.0(2)	3364.7(2)	13.66(5)
N1	2818.9(15)	6932.1(14)	3203.3(15)	16.3(8)
N2	3301.0(13)	7590.0(14)	2974.5(13)	13.1(7)
N3	1704.5(15)	6449.0(16)	4342.0(14)	19.5(8)
N4	2399.4(16)	6784.9(17)	4383.4(15)	22.7(9)
O1	1325(3)	8675(3)	3675(3)	91(4)
O2	1046(9)	8284(7)	4536(7)	179(9)
Si1	4111.7(6)	6386.4(6)	2720.1(7)	30.0(4)
C31	20(8)	7796(7)	5028(7)	109(8)
O3	105(6)	8172(7)	4731(6)	156(8)

Table S22. Anisotropic Displacement Parameters (Å^2×10^3) for $C_{89.41875}H_{141.67625}Cl_3Ir_3N_{12}O_5$.. The anisotropic displacement factor exponent takes the form: $-2\pi^2[h^2a^{*2}U_{11}+2hka^*b^*U_{12}+…]$.

Atom	U_{11}	U_{22}	U_{33}	U_{23}	U_{13}	U_{12}
C1	21(2)	14(2)	18(2)	1.0(17)	-0.5(18)	-1.6(18)
C2	19(2)	19(2)	24(2)	-0.7(19)	3.2(19)	0.1(18)
C3	13(2)	17(2)	24(2)	-1.2(18)	1.7(18)	0.9(17)
C4	17(2)	17(2)	16(2)	-2.5(17)	0.7(17)	-0.8(17)
C5	39(4)	37(4)	129(9)	-35(5)	-15(5)	8(3)
C6	38(4)	59(5)	66(5)	-16(4)	-15(4)	19(4)
C7	60(5)	68(6)	87(7)	16(5)	44(5)	24(5)
C8	17(2)	13.5(19)	14.8(19)	1.3(16)	-0.8(16)	-3.2(17)
C9	25(3)	43(3)	11(2)	1(2)	-2.1(18)	-11(2)
C10	33(3)	52(4)	15(2)	0(2)	-1(2)	-16(3)
C11	19(2)	24(2)	13(2)	0.3(18)	3.1(17)	-5.2(19)
C12	25(2)	27(3)	15(2)	-1.4(19)	4.2(19)	-3(2)
C13	34(3)	27(3)	21(2)	-6(2)	10(2)	-16(2)
C14	19(2)	44(3)	21(2)	-9(2)	8(2)	-13(2)
C15	19(2)	42(3)	23(3)	-5(2)	1(2)	-2(2)
C16	20(2)	27(3)	15(2)	-3.9(18)	4.5(18)	-4.1(19)
C17	41(3)	20(2)	23(2)	3(2)	6(2)	0(2)
C18	30(3)	64(5)	40(4)	-22(3)	9(3)	-21(3)
C19	33(3)	23(3)	29(3)	-2(2)	-1(2)	1(2)
C20	20(2)	28(3)	12(2)	1.9(18)	-2.8(17)	-7(2)
C21	18(2)	26(3)	19(2)	0.8(19)	-1.6(18)	-2.1(19)
C22	22(2)	21(2)	19(2)	-1.1(18)	0.7(19)	-4.5(19)
C23	19(2)	24(2)	16(2)	-0.6(18)	-0.1(18)	-3.2(19)
C24	17(2)	26(3)	26(3)	0(2)	0.4(19)	0.4(19)
C25	23(2)	25(2)	20(2)	0.0(19)	-3.6(19)	-4(2)
C26	20(3)	39(3)	44(4)	-5(3)	-1(2)	3(2)
C27	21(2)	30(3)	33(3)	-3(2)	3(2)	-7(2)
C28	40(3)	24(3)	41(3)	2(3)	-5(3)	-4(2)
C29	54(3)	54(3)	54(3)	-2(4)	2(4)	2(4)
C30	132(16)	155(19)	39(7)	-17(9)	-17(8)	78(14)
Cl1	23.2(5)	14.1(5)	19.4(5)	2.8(4)	-0.5(4)	-3.9(4)
Ir1	14.43(8)	13.82(8)	12.73(8)	1.78(6)	0.39(6)	-1.71(6)
N1	17.3(19)	13.5(17)	18.2(18)	0.2(15)	0.0(15)	-1.0(14)
N2	11.3(10)	15.4(10)	12.5(10)	2.8(8)	-2.0(8)	-2.7(8)
N3	20(2)	26(2)	12.3(17)	0.1(16)	0.3(15)	-6.6(17)
N4	24(2)	32(2)	12.2(18)	5.1(17)	-0.6(16)	-9.6(18)
O1	91(4)	91(4)	91(4)	-12(5)	12(5)	12(5)
O2	220(20)	144(17)	171(18)	19(13)	52(16)	-28(14)
Si1	20.9(7)	24.5(8)	44.6(10)	-5.5(7)	5.1(7)	3.7(6)
C31	121(17)	117(16)	88(13)	50(12)	22(11)	20(13)
O3	123(14)	173(17)	172(17)	68(14)	-25(12)	-29(12)

Table S23. Bond lengths for $C_{89.41875}H_{141.67625}Cl_3Ir_3N_{12}O_5$.

Atom	Atom	Length/Å	Atom	Atom	Length/Å
C1	C2	1.392(7)	C14	C15	1.403(9)
C1	N1	1.346(6)	C14	C18	1.513(8)
C2	C3	1.393(7)	C15	C16	1.396(7)
C2	Si1	1.888(5)	C16	C19	1.505(8)
C3	N2	1.313(6)	C20	C21	1.397(8)
C4	N1	1.333(6)	C20	C25	1.397(8)
C4	N2	1.357(6)	C20	N4	1.430(6)
C5	Si1	1.862(8)	C21	C22	1.384(7)
C6	Si1	1.858(8)	C21	C26	1.517(8)
C7	Si1	1.846(9)	C22	C23	1.398(7)
C8	Ir1	1.962(5)	C23	C24	1.396(7)
C8	N3	1.375(6)	C23	C27	1.507(7)
C8	N4	1.358(6)	C24	C25	1.387(7)
C9	C10	1.517(8)	C25	C28	1.507(8)
C9	N3	1.473(6)	C29	O1	1.37(2)
C10	N4	1.463(7)	C30	O2	1.42(2)
C11	C12	1.398(7)	Cl1	Ir1	2.5250(12)
C11	C16	1.399(7)	Ir1	N1	2.243(4)
C11	N3	1.425(6)	Ir1	N2^{1}	2.177(4)
C12	C13	1.399(8)	N2	Ir1^{2}	2.177(4)
C12	C17	1.511(8)	C31	O3	1.36(2)
C13	C14	1.379(9)			

Table S24. Torsion angles for $C_{89.41875}H_{141.67625}Cl_3Ir_3N_{12}O_5$.

A	B	C	D	Angle/°	A	B	C	D	Angle/°
C1	C2	C3	N2	0.0(8)	C21	C22	C23	C24	-1.7(8)
C1	C2	Si1	C5	25.7(6)	C21	C22	C23	C27	176.4(5)
C1	C2	Si1	C6	-93.0(6)	C22	C23	C24	C25	1.3(8)
C1	C2	Si1	C7	147.8(6)	C23	C24	C25	C20	0.6(8)
C2	C1	N1	C4	0.0(7)	C23	C24	C25	C28	-179.9(5)
C2	C1	N1	Ir1	168.0(4)	C25	C20	C21	C22	1.8(8)
C2	C3	N2	C4	1.0(7)	C25	C20	C21	C26	-177.2(5)
C2	C3	N2	Ir1[1]	-179.1(4)	C25	C20	N4	C8	-93.7(7)
C3	C2	Si1	C5	-157.8(6)	C25	C20	N4	C10	85.8(7)
C3	C2	Si1	C6	83.5(6)	C26	C21	C22	C23	179.2(5)
C3	C2	Si1	C7	-35.7(7)	C27	C23	C24	C25	-176.7(5)
C9	C10	N4	C8	-14.4(7)	Ir1	C8	N3	C9	-172.9(4)
C9	C10	N4	C20	166.1(5)	Ir1	C8	N3	C11	-0.8(8)
C10	C9	N3	C8	-15.9(6)	Ir1	C8	N4	C10	-174.7(4)
C10	C9	N3	C11	171.0(5)	Ir1	C8	N4	C20	4.8(8)
C11	C12	C13	C14	0.4(8)	N1	C1	C2	C3	-0.5(8)
C12	C11	C16	C15	-4.3(7)	N1	C1	C2	Si1	176.2(4)
C12	C11	C16	C19	175.9(5)	N1	C4	N2	C3	-1.6(7)
C12	C11	N3	C8	104.5(6)	N1	C4	N2	Ir1[1]	178.5(4)
C12	C11	N3	C9	-83.7(6)	N2	C4	N1	C1	1.1(7)
C12	C13	C14	C15	-3.3(8)	N2	C4	N1	Ir1	-167.1(3)
C12	C13	C14	C18	174.9(5)	N3	C8	N4	C10	4.9(6)
C13	C14	C15	C16	2.5(8)	N3	C8	N4	C20	-175.6(5)
C14	C15	C16	C11	1.3(8)	N3	C9	C10	N4	16.6(6)
C14	C15	C16	C19	-179.0(5)	N3	C11	C12	C13	174.9(4)
C16	C11	C12	C13	3.6(7)	N3	C11	C12	C17	-3.8(7)
C16	C11	C12	C17	-175.1(5)	N3	C11	C16	C15	-175.5(4)
C16	C11	N3	C8	-84.1(7)	N3	C11	C16	C19	4.8(7)
C16	C11	N3	C9	87.7(6)	N4	C8	N3	C9	7.5(6)
C17	C12	C13	C14	179.0(5)	N4	C8	N3	C11	179.6(5)
C18	C14	C15	C16	-175.7(5)	N4	C20	C21	C22	175.7(4)
C20	C21	C22	C23	0.2(8)	N4	C20	C21	C26	-3.2(7)
C21	C20	C25	C24	-2.2(8)	N4	C20	C25	C24	-176.0(5)
C21	C20	C25	C28	178.2(5)	N4	C20	C25	C28	4.4(8)
C21	C20	N4	C8	92.3(7)	Si1	C2	C3	N2	-176.7(4)
C21	C20	N4	C10	-88.3(6)					

Table S25. Atomic occupancy for $C_{89.41875}H_{141.67625}Cl_3Ir_3N_{12}O_5$.

Atom	*Occupancy*	Atom	*Occupancy*	Atom	*Occupancy*
H29A	0.3333	H29B	0.3333	H29C	0.3333
C30	0.75(2)	H30A	0.75(2)	H30B	0.75(2)
H30C	0.75(2)	H1A	0.3333	O2	0.75(2)
H2	0.75(2)	C31	0.722(19)	H31A	0.722(19)
H31B	0.722(19)	H31C	0.722(19)	O3	0.722(19)
H3A	0.722(19)				

Table S26. Hydrogen Atom Coordinates (Å×10[4]) and Isotropic Displacement Parameters (Å[2]×10[3]) for $C_{89.41875}H_{141.67625}Cl_3Ir_3N_{12}O_5$.

Atom	*x*	*y*	*z*	U(eq)
H1	3128	6311	3092	22
H3	3925	7427	2722	22
H4	2655	7608	3258	20
H5A	3664	5807	2300	103
H5B	3667	5680	2846	103
H5C	4114	5564	2529	103
H6A	4351	6179	3501	82
H6B	4600	6657	3351	82
H6C	4796	6168	3161	82
H7A	4542	6956	2279	107
H7B	4243	6636	1930	107
H7C	4721	6442	2146	107
H9A	1704	6248	5042	31
H9B	1565	6791	4969	31
H10A	2341	6948	5087	40
H10B	2487	6409	4997	40
H13	734	5213	4132	33
H15	171	6510	3918	33
H17A	1548	5107	4333	34
H17B	1715	5503	4692	34
H17C	1901	5507	4164	34
H18A	-71	5518	3503	67
H18B	-342	5809	3898	67
H18C	-133	5304	4017	67
H19A	618	7215	4027	42
H19B	1155	7168	3877	42
H19C	1015	7172	4420	42
H22	3256	8107	4235	25
H24	3963	6865	4118	28
H26A	2415	8136	4322	51
H26B	2264	7755	4704	51
H26C	2123	7684	4166	51
H27A	4146	8085	4249	42
H27B	4152	7886	3725	42
H27C	4408	7603	4135	42
H28A	3008	6121	4027	52
H28B	3203	6126	4552	52
H28C	3560	6121	4120	52
H29A	1938	8469	3447	81
H29B	1531	8447	3062	81
H29C	1553	8062	3469	81
H30A	1377	8181	5146	163
H30B	1149	8690	5099	163
H30C	1621	8567	4823	163
H	1920(20)	7120(20)	3400(20)	12(13)
HA	1590(30)	6510(30)	3400(30)	36(19)
H1A	1263	8534	3925	137
H2	843	8093	4627	268
H31A	166	7513	4899	163
H31B	-318	7747	5057	163
H31C	153	7862	5337	163
H3A	-72	8394	4803	234

2.9 Complex characterisation by NMR

The NMR data for species **2** and **3** is detailed below. These reflect the dominant forms of the SABRE catalyst.

Table S27: NMR characterisation data for 2a in methanol-d_4 at 245 K

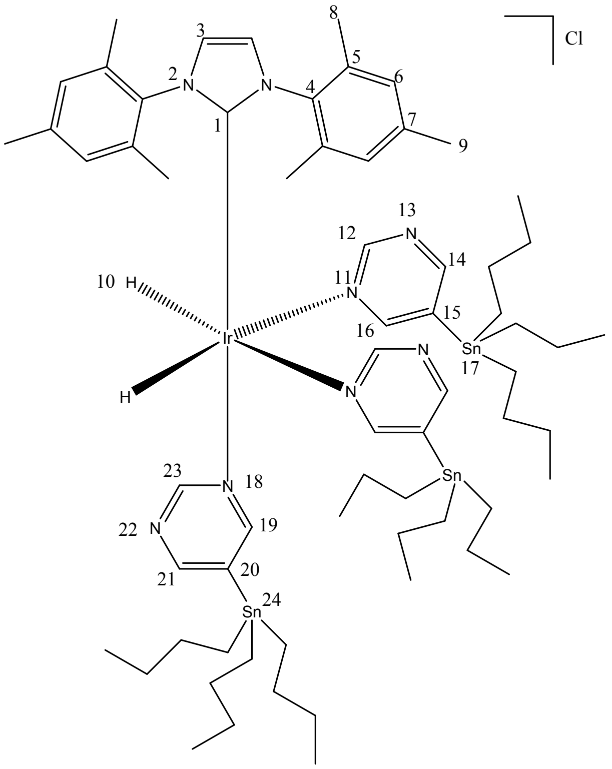

Resonance number	^{1}H (ppm)	^{13}C (ppm)	^{15}N (ppm) or ^{119}Sn (ppm)
1		150.5	
2			194.6
3	7.33	123.0	
4		137.3	
5		135.0	
6	6.73	128.5	
7		137	
8	2.08	17.64	
9	2.21	20.10	
10	-22.06		
11			241.4
12	9.03	162.02	
13			295.4
14	8.85	162.76	
15		136.6	
16	8.30	165.47	
17			-50.14
18			228
19	8.44	168.51	
20		136.6	
21	8.75	163.0	
22			297.9
23	8.37	161.92	
24			-50.60

Table S28 NMR characterisation data for 2b in methanol-d_4 at 245 K

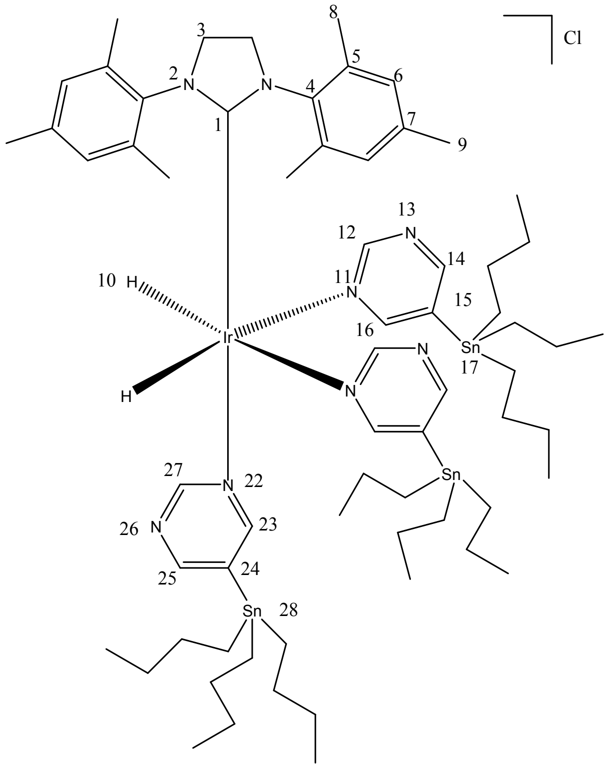

Resonance number	^{1}H (ppm)	^{13}C (ppm)	^{15}N (ppm) or ^{119}Sn (ppm)
1		163.44	
2			194.65
3	3.96		
4			
5		135.89	
6	6.63	168.76	
7		137.51	
8	2.27	17.58	
9	2.16	20.03	
10	-22.15		
11			240.56
12	9.06	162.16	
13			294.70
14	8.85	157.09	
15			
16	8.23	165.28	
17			-49.92
18			226.70
19	8.35	167.99	
20			
21	8.73	163.14	
22			295.15
23	8.26	161.4	
28			-50.44

Table S29 NMR characterisation data for 3a in methanol-d_4 at 245 K

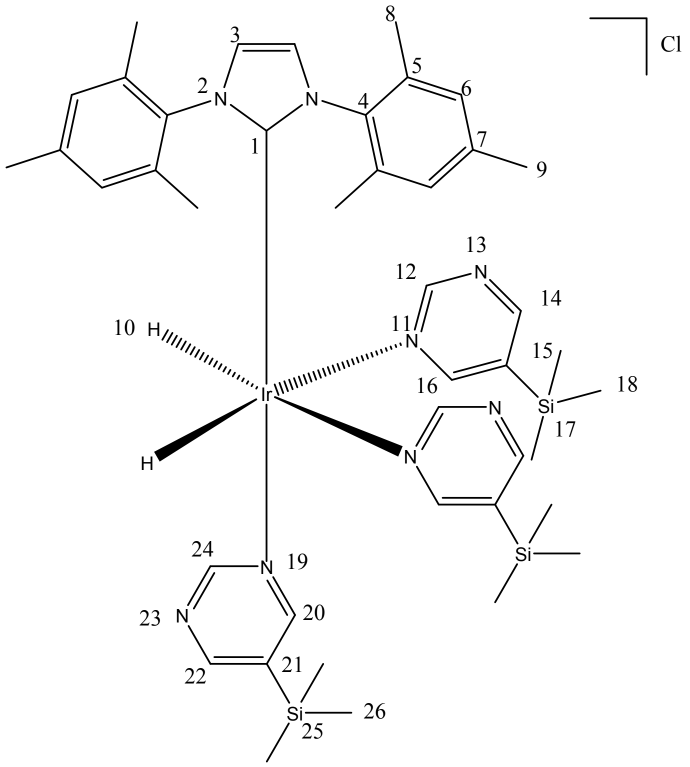

Resonance number	^{1}H (ppm)	^{13}C (ppm)	^{15}N (ppm) or ^{29}Si (ppm)
1		150.6	
2			194.8
3	7.34	123.0	
4		137.1	
5		135.1	
6	6.75	128.6	
7		138.5	
8	2.08	17.55	
9	2.21	19.97	
10	-22.09		
11			240.6
12	9.12 s	162.73	
13			294.7
14	8.95	163.67	
15		134.1	
16	8.44 d 2.0 Hz	163.66	
17			-3.46
18	0.26	-3.2	
19			226.9
20	8.82	160.81	
21		134.3	
22	7.86 d	164.24	
23			295.1
24	8.99	164.2	
25			-3.67
26	0.08	-3.2	

Table S30 NMR characterisation data for 3b in methanol-d_4 at 245 K

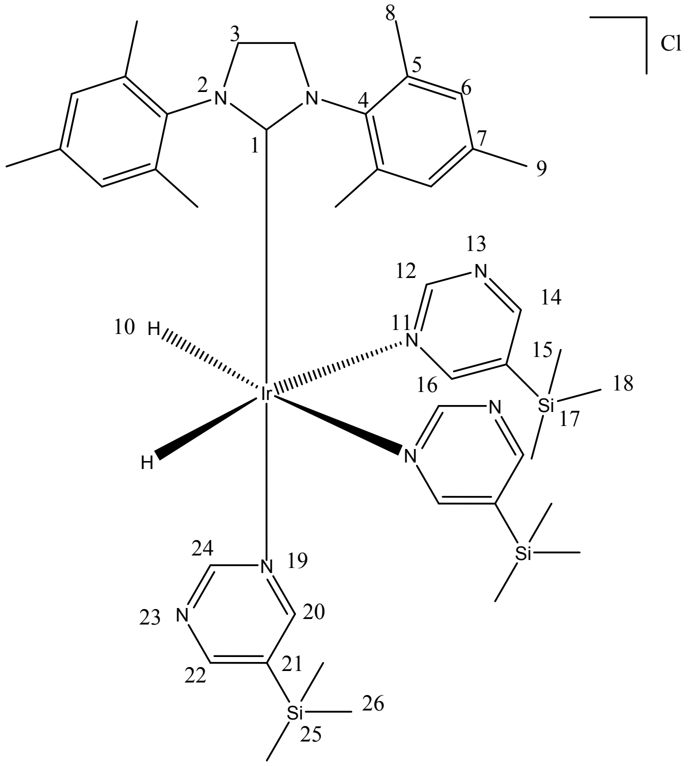

Resonance number	^{1}H (ppm)	^{13}C (ppm)	^{15}N (ppm) or ^{29}Si (ppm)
1		170.7	
2			-
3	3.97	78.5	
4		137.5	
5		135.8	
6	6.66	128.7	
7		137.8	
8	2.28	17.57	
9	2.15	19.89	
10	-22.09		
11			240.5
12	9.12 s	162.7	
13			294.6
14	8.95	160.7	
15		134.17	
16	8.36 br	163.55	
17			-3.4
18	0.25	-3.2	
19			228.5
20	8.81	160.9	
21		134.2	
22	7.68 br	163.3	
23			295.0
24	8.92	164.3	
25			-3.67
26	0.04	-3.42	

3. References

1. Torres, O., M. Martín, and E. Sola, *Labile N-Heterocyclic Carbene Complexes of Iridium.* Organometallics, 2009. **28**(3): p. 863-870.
2. Stott, K., et al., *Excitation Sculpting in High-Resolution Nuclear Magnetic Resonance Spectroscopy: Application to Selective NOE Experiments.* Journal of the American Chemical Society, 1995. **117**(14): p. 4199-4200.
3. Stonehouse, J., et al., *Ultrahigh-Quality NOE Spectra.* Journal of the American Chemical Society, 1994. **116**(13): p. 6037-6038.
4. Shaw, A.A., et al., *Artifact-Free PFG-Enhanced Double-Quantum-Filtered COSY Experiments.* Journal of Magnetic Resonance, Series A, 1996. **120**(1): p. 110-115.
5. Ancian, B., et al., *Artifact-Free Pure Absorption PFG-Enhanced DQF-COSY Spectra Including a Gradient Pulse in the Evolution Period.* Journal of Magnetic Resonance, 1997. **125**(2): p. 348-354.
6. Kessler, H., et al., *Transformation of homonuclear two-dimensional NMR techniques into one-dimensional techniques using Gaussian pulses.* Journal of Magnetic Resonance (1969), 1986. **70**(1): p. 106-133.
7. Vassilev, N.G. and V.S. Dimitrov, *Dynamic NMR: combined use of 1D selective EXSY and complete lineshape analysis of spectra subjected to reference deconvolution and linear prediction or the maximum entropy method.* Magnetic Resonance in Chemistry, 2001. **39**(10): p. 607-614.
8. Butcher, J.C., *Numerical Methods for Ordinary Differential Equations*. 2 ed. 2008, New York: John Wiley & Sons.
9. Runge, C., *Ueber die numerische Auflösung von Differentialgleichungen.* Mathematische Annalen, 1895. **46**(2): p. 167-178.
10. Kutta, W., *Beitrag zur näherungsweisen Integration totaler Differentialgleichungen.* Z.Math.Phys., 1901. **46**: p. 435-453.
11. Levenberg, K., *A Method for the Solution of Certain Non-Linear Problems in Least Squares.* The Quarterly of Applied Mathematics, 1944(2): p. 164-168.
12. Marquardt, D., *An Algorithm for Least-Squares Estimation of Nonlinear Parameters.* Journal of the Society for Industrial and Applied Mathematics, 1963. **11**(2): p. 431-441.
13. Polanyi, J.C., *Concepts in reaction dynamics.* Accounts of Chemical Research, 1972. **5**(5): p. 161-168.
14. Eyring, H., *The Activated Complex and the Absolute Rate of Chemical Reactions.* Chemical Reviews, 1935. **17**(1): p. 65-77.
15. Lloyd, L.S., et al., *Hyperpolarisation through reversible interactions with parahydrogen.* Catalysis Science & Technology, 2014. **4**(10): p. 3544-3554.
16. Dolomanov, O.V., et al., *OLEX2: a complete structure solution, refinement and analysis program.* Journal of Applied Crystallography, 2009. **42**(2): p. 339-341.
17. Sheldrick, G., *A short history of SHELX.* Acta Crystallographica Section A, 2008. **64**(1): p. 112-122.
18. Sheldrick, G., *Crystal structure refinement with SHELXL.* Acta Crystallographica Section C, 2015. **71**(1): p. 3-8.
